# Metastatic brain tumors: from development to cutting‐edge treatment

**DOI:** 10.1002/mco2.70020

**Published:** 2024-12-20

**Authors:** Guilong Tanzhu, Liu Chen, Jiaoyang Ning, Wenxiang Xue, Ce Wang, Gang Xiao, Jie Yang, Rongrong Zhou

**Affiliations:** ^1^ Department of Oncology Xiangya Hospital Central South University Changsha China; ^2^ NHC Key Laboratory of Radiobiology School of Public Health Jilin University Changchun Jilin China; ^3^ Department of Radiology China‐Japan Friendship Hospital Beijing China; ^4^ Department of Dermatology Xiangya Hospital Central South University Changsha China; ^5^ Xiangya Lung Cancer Center Xiangya Hospital Central South University Changsha China; ^6^ National Clinical Research Center for Geriatric Disorders Xiangya Hospital Central South University Changsha Hunan Province China

**Keywords:** diagnosis and treatment, metastatic brain tumors, molecular mechanisms, multiomics, tumor microenvironment

## Abstract

Metastatic brain tumors, also called brain metastasis (BM), represent a challenging complication of advanced tumors. Tumors that commonly metastasize to the brain include lung cancer and breast cancer. In recent years, the prognosis for BM patients has improved, and significant advancements have been made in both clinical and preclinical research. This review focuses on BM originating from lung cancer and breast cancer. We briefly overview the history and epidemiology of BM, as well as the current diagnostic and treatment paradigms. Additionally, we summarize multiomics evidence on the mechanisms of tumor occurrence and development in the era of artificial intelligence and discuss the role of the tumor microenvironment. Preclinically, we introduce the establishment of BM models, detailed molecular mechanisms, and cutting‐edge treatment methods. BM is primarily treated with a comprehensive approach, including local treatments such as surgery and radiotherapy. For lung cancer, targeted therapy and immunotherapy have shown efficacy, while in breast cancer, monoclonal antibodies, tyrosine kinase inhibitors, and antibody–drug conjugates are effective in BM. Multiomics approaches assist in clinical diagnosis and treatment, revealing the complex mechanisms of BM. Moreover, preclinical agents often need to cross the blood–brain barrier to achieve high intracranial concentrations, including small‐molecule inhibitors, nanoparticles, and peptide drugs. Addressing BM is imperative.

## Graphical abstract

This abstract briefly reviews the diagnosis and treatment of brain metastases from lung cancer and breast cancer, the progress of artificial intelligence and multiomics. Importantly, we focus on the pathogenesis of brain metastases and the tumor microenvironment to seek treatment strategies.

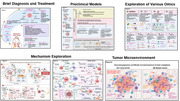

## 1 INTRODUCTION

Metastatic brain tumors, also called brain metastases (BM), are a common complication of advanced tumors with a poor prognosis, representing a major clinical challenge in tumor treatment.^1^ With advancements in primary tumor therapies and imaging technology, the survival of patients has been prolonged, leading to an increase in the number of patients diagnosed with BM. Primary tumors that commonly metastasize to the brain include lung cancer (LC), breast cancer (BC), and melanoma. Data from the National Cancer Database reveal that among patients with newly diagnosed BMs, the proportions for non‐small cell lung cancer (NSCLC), small cell lung cancer (SCLC), melanoma, and BC are 16.0, 10.3, 1.5, and 0.3%, respectively.^2^

LC is the second most common and the leading malignant tumor in terms of morbidity and mortality, respectively. It can be classified into SCLC and NSCLC, with NSCLC accounting for about 85% of cases.^3^ Distant metastasis is a leading cause of death in advanced NSCLC patients while the brain is the most common site. Approximately 30% of NSCLC patients present with BM at initial diagnosis, and as the disease progresses, about 60% of patients will eventually develop BM.^4^ The median overall survival (OS) of untreated NSCLC BM patients is only 4–9 months. In contrast, BC patients, due to their long survival with median OS up to 28 years, have nearly a 50% chance of developing BM in the later stages of the disease.^5^ The incidence of BC brain metastasis (BCBM) ranks second among various primary tumors. However, the proportion of newly diagnosed BM patients is relatively low.^6^ Data from 10‐year follow‐ups indicate that triple‐negative, HER2‐positive (HER2+), and HR+/HER2‐negative (HER2−) BC subtypes are more likely to develop BM.^6^, ^7^, ^8^ The prognosis becomes extremely poor once BM occurs, with a median survival of approximately 10 months.^6^, ^9^ Metachronous BMs are the most common, occurring in approximately 60% of cases, usually within 2 years of the primary tumor diagnosis.^10^ In comparison, LC progresses more rapidly than BC and tends to develop BMs in a shorter time.^10^ For instance, the time from tumor diagnosis to the occurrence of BMs varies significantly between primary tumors, with LC showing a median interval of 5.3 months compared with 44.4 months for BC.^7^, ^11^

In the 1970s, studies on brain surgical specimens and autopsies indicated that the incidence of BM ranged from approximately 2.8 to 11.1 per 100,000 individuals.^12^, ^13^, ^14^ However, these estimates may have been an underrepresentation due to limitations in medical technology at that time.^15^ In 1978, Posner and Chernik^16^ conducted autopsies on 2375 cases and found that 24% of tumor patients had intracranial metastases. Additionally, about two‐thirds of patients diagnosed with BM through biopsy exhibited neurological symptoms. With the decline in autopsy rates and advancements in medical technology, noninvasive methods for detecting BM have become increasingly prevalent. Lokich^17^ highlighted the importance of computed tomography (CT) as a noninvasive diagnostic tool for central nervous system metastases. CT can provide detailed information on the ventricular system, accurately depict the number and size of lesions, assess the extent of secondary cerebral edema.^17^, ^18^ Despite these capabilities, the sensitivity of CT for detecting small lesions remains relatively low, even with the use of iodine‐based contrast agents.^19^ In the 1980s, magnetic resonance imaging (MRI) began to replace CT as the preferred imaging modality for diagnosing BM and evaluating treatment efficacy.^20^ According to Suh et al.,^21^ MRI has become the cornerstone of radiologic evaluation due to its superior ability to visualize small parenchymal metastases and leptomeningeal involvement compared with CT. Larkin et al.^22^ further demonstrated that multimodal MRI, particularly gadolinium‐enhanced T1‐weighted images, offers the highest sensitivity and accuracy for detecting smaller metastatic lesions. Despite the advantages of MRI, CT remains relevant due to its availability, cost‐effectiveness, and efficiency in screening for various conditions. Positron emission tomography (PET), introduced in the 1970s, complements MRI and CT by providing metabolic information about BM and other abnormalities.^23^, ^24^ Brooks et al.^25^ utilized technetium Tc 99 m radionuclide scanning in the 1970s to identify 75% of intracranial metastatic lesions accurately and to differentiate vascular lesions through sequential examinations. However, the sensitivity and specificity of fludeoxyglucose (FDG) PET for detecting BM are lower than those of MRI. Amino acid PET tracers, which do not rely on the disruption of the blood–brain barrier (BBB) for absorption, have shown superior diagnostic performance compared with FDG PET and MRI‐based perfusion and diffusion‐weighted imaging.^26^, ^27^, ^28^

In recent years, the prognosis of LCBM and BCBM has improved significantly, and related research has made great achievements both clinically and preclinically. In this review, we briefly trace the history and epidemiology of BM. Next, we summarize the current diagnostic and treatment paradigms for BM arising from LC and BC. In the era of artificial intelligence (AI), technologies such as imaging omics and machine learning have significantly advanced the diagnosis and treatment of BM, prompting us to focus on the progress of these technologies and research directions. The treatment of BM is currently comprehensive.^29^ We emphasize the latest progress in various treatment methods including surgery, radiotherapy (RT), chemotherapy, immunotherapy, antibody–drug conjugates (ADCs), and targeted therapy. We also address the challenges encountered in comprehensive treatment and look forward to the development of new technologies. The occurrence of BM is extremely complex, and its mechanisms have not yet been fully clarified.^1^ With the development of biological science and technology, multiomics has become an important method. The application of genomics, transcriptomics (including bulk RNA transcriptome, single‐cell transcriptome, and spatial transcriptome), and proteomics has provided insights into the biological characteristics of BM.^30^, ^31^, ^32^ BM is a multistage, multistep pathological process, including local invasion of tumor cells from the primary site, intravasation into the blood or lymphatic vessels, survival in the circulation, penetration, and extravasation of the BBB, and intracranial colonization and regrowth.^33^, ^34^ Most importantly, we present the latest findings on the entire process of BM. We examine the tumor microenvironment (TME) of BM and focus on therapeutic targets based on the developmental process of BM and the TME. This review highlights the latest progress in LCBM and BCBM (mainly brain parenchymal metastasis), summarizing the developmental mechanisms and cutting‐edge treatments, aiming to provide researchers with comprehensive and in‐depth insights.

## 2 DIAGNOSIS OF METASTATIC BRAIN TUMORS

**FIGURE 1:**
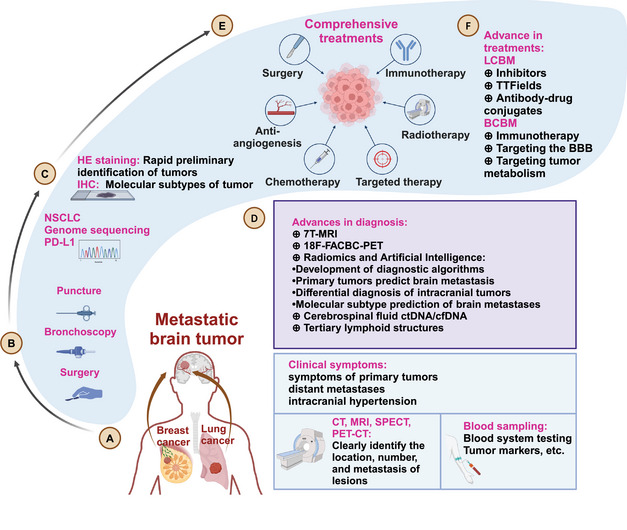
Diagnosis and treatment of metastatic brain tumors. (A) Brain metastasis from lung cancer and breast cancer can manifest as clinical symptoms, distant metastasis, and intracranial hypertension; laboratory tests such as blood tumor markers and imaging examinations can be used for auxiliary diagnosis and characterization of metastatic lesions. (B) The diagnostic approach for brain metastasis includes surgery, fiberoptic bronchoscopy, and puncture biopsy. (C) HE staining and IHC are often used to confirm the primary tumor source and classify brain metastasis. For brain metastasis from NSCLC, genomic sequencing and PD‐L1 testing guide the treatment. (D) Advances in the diagnosis of brain metastasis have been driven by technologies such as 7T‐MRI, 18‐FACBC PET, imaging genomics, artificial intelligence, tertiary lymphoid structures, and liquid biopsy like ctDNA/cfDNA. (E) Treatments of brain metastasis typically involve a combination of surgical treatment, immunotherapy, antiangiogenic therapy, radiotherapy, chemotherapy, and targeted therapy. (F) Advances in the treatments include inhibitors, TTFields, antibody–drug conjugates for lung cancer brain metastasis and immunotherapy, strategies like targeting the BBB or tumor metabolism for breast cancer brain metastasis.

With the advancement of medical technology, the diagnosis and treatment of BM have made great progress (Figure 1). The diagnosis of BM includes molecular pathology and imaging examinations. The gold standard for diagnosis is obtaining tissue samples through surgery or biopsy for molecular pathology testing. When combined with immunohistochemistry and genetic testing, the primary tumors of BM can be identified. Common indicators for the origin of SCLC include: proGRP, Syn, NSE, CgA, CD56, CEA, and TTF‐1. For NSCLC, common indicators include: TTF‐1, Napsin A, CK5/6, P63, and P40. In BC, common indicators are E‐cad, P120, P63, CK5/6, ER, PR, HER‐2, TOPO2A, and androgen receptor (AR).^34^, ^35^ Additionally, driver gene mutations and PD‐(L)1 levels are assessed in NSCLC patients. Serum tumor markers also assist in the diagnosis and treatment of BM.^36^ For patients suspected of leptomeningeal metastasis, cerebrospinal fluid (CSF) testing can be performed through lumbar puncture.^37^, ^38^

MRI is a commonly used screening and treatment assessment method for BM patients. CT can serve as an adjunct for patients who are not suitable for MRI.^39^ Patients with large BM lesions often experience symptoms such as intracranial hypertension (headache, nausea or vomiting, epilepsy, or neurological deficits) and are referred to neurosurgery for treatment, after undergoing diagnosis by MRI, CT, and other examinations. Asymptomatic BM patients are often discovered during follow‐up examinations. After primary tumor is diagnosed, brain MRI is routinely performed every 3–6 months.^40^, ^41^ MRI is highly sensitive for lesions smaller than 5 mm.

BMs from different primary tumors exhibit distinct intracranial distributions.^42^ Bonert et al.^43^ employed deep learning models to compare the BMs from various primary tumors and discovered significant differences in the anatomical distribution of BMs between BC, LC or kidney cancer. Conversely, the distribution patterns of LC, kidney cancer and melanoma, were found to be similar.^44^ BC, LC, and colorectal cancer commonly metastasize to more posterior/caudal neuroanatomical regions, particularly the cerebellum.^42^ LCBM are mainly found in the white matter, cerebellar hemispheres, and middle frontal gyrus, but are less common in the inferior frontal gyrus and temporal pole of the frontal lobe.^45^ Shi et al.^45^ described the differences in the spatial distribution of BM from SCLC and NSCLC. The precentral gyrus, middle frontal gyrus, paracentral lobule, and cerebellar hemispheres are high‐risk areas for BM from NSCLC.^45^ Similarly, atlas analysis indicated that the low‐risk area for BM from SCLC is the inferior frontal gyrus of the frontal lobe, and the high‐risk area is the cerebellar hemisphere.^45^, ^46^ Lung adenocarcinoma (LUAD) predominantly affects the frontal lobe, whereas squamous cell carcinoma of the lung is more likely to be found in the cerebellum.^42^ By integrating MRI with AI, Han et al.^47^ investigated the anatomical distribution of intracranial lesions in BCBM patients and identified the cerebellum, occipital lobe, and thalamus as higher‐risk areas for BCBM. Notably, triple‐negative breast cancer (TNBC) patients were at increased risk for lesions in the hippocampus and brainstem.^47^ More precisely, Neman et al.^48^ analyzed the distribution of lesions in 2106 patients with BMs and found that LC, BC, and melanoma were prone to metastasize to the bilateral temporal lobes, the right cerebellar hemisphere, and the left temporal lobe, respectively.

Different MRI techniques exhibit different sensitivities and specificities.^49^ With the advancement of MRI technology, the advantages of a commercial 7‐T MRI scanner, such as improved spatial resolution, increased signal‐to‐noise ratio, and increased contrast‐to‐noise ratio, have assisted in the diagnosis and treatment evaluation of brain tumors.^50^, ^51^ Longitudinal GRASP dynamic contrast‐enhanced MRI can distinguish BM progression from radiation effects after stereotactic radiosurgery (SRS).^52^ Radiomics and AI can not only distinguish LCBM from primary intracranial tumors, but also differentiate BM originating from different primary tumors and predict LC driver gene mutations.^53^ Conventional PET–CT is relatively insensitive to brain tumors, but more advanced imaging techniques may have added value.^39^, ^54^ Although 18F‐FACBC PET/MRI cannot improve the detection rate of BM, it has some ability to distinguish the BM source of primary tumors.^55^ Furthermore, PET–CT was employed to assess HER2 expression in BCBM patients. The maximum standardized uptake values (SUV_max_) of 18F‐fluorodeoxyglucose PET were significantly higher in HER2+ patients compared with HER2− patients.^56^

Liquid biopsy is crucial for the diagnosis, prognostic stratification, prediction of treatment response, and detection of tumor progression in BM patients.^57^, ^58^ With the advancement of liquid biopsy, cell‐free DNA (cfDNA)and circulating tumor DNA (ctDNA) have been used in the diagnosis and treatment of primary tumors.^59^, ^60^ CfDNA is released by normal cells and cells exhibiting pathological processes (e.g., inflammation and tumors). ctDNA is a subset of cfDNA released by tumor cells through a combination of apoptosis, necrosis, and secretion.^36^, ^61^ Chen et al.^59^ used ctDNA for postoperative monitoring of LC. Patients with colorectal cancer BM have higher cfDNA levels than healthy people.^62^ In detecting the genome, the results show that CSF ctDNA can more comprehensively reflect the mutation status of BM than plasma ctDNA. Minor allele frequency is highly correlated with BM tumor size (*R* = 0.95), and CSF circulating tumor cells (CTC) has a faithful mutation allele frequency correlation and is much higher than the mutation detection rate of plasma CTC (83.33 vs. 27.78%).^63^, ^64^ A machine learning model for early diagnosis of BM based on CSF ctDNA has been developed.^65^ In BM, the detection rate of CSF cfDNA is significantly higher than that of plasma cfDNA, and the abundance of cfDNA is significantly reduced after RT, but there is no significant change in CSF TMB.^66^ The combination of ctDNA and T cell repertoire can predict the efficacy of RT for BM.^61^ Prospective clinical studies have also demonstrated the bright future of CSF CTCs.^67^ In HER2+ BC patients, detecting FGFR aberrations in ctDNA often indicates an increased risk of BM.^68^ The advancement of liquid biopsy has been significantly driven by the detection of genomic alterations through ctDNA. Alder et al.^69^ analyzed serum ctDNA from 253 patients with BM, finding that ESR1 and BRCA2 mutations were more prevalent in BCBM patients. Although sequencing of eight brain tissues and corresponding ctDNA revealed a high mutation consistency (seven out of eight), the larger and prospective studies are needed to validate the diagnostic feasibility of ctDNA.^69^ Similarly, Curtaz et al.^70^ identified miR‐576‐3p and miR‐130a‐3p in exosomes from blood samples, achieving area under curve (AUC) values of 0.705 and 0.699, respectively, for predicting BM occurrence.

Tertiary lymphoid structures (TLS) are organized immune cell aggregates within the TME that resemble secondary lymphoid organs. These structures are closely associated with immunotherapy responses and prognosis across various cancers.^71^ Notably, Zhao et al.^72^ assessed TLS in BCBM patients and found that high TLS density was present in about half of the patients, correlating with longer OS and progression‐free survival (PFS). Additionally, they integrated factors such as age, systemic chemoradiotherapy, tumor molecular subtype, and Karnofsky Performance Status with the TLS score to develop a nomogram for predicting the clinical prognosis of BCBM patients, thereby facilitating the clinical application of TLS.^72^

### 2.1 The application of new technologies in the era of AI

With the development of science and technology, the integration of medicine and engineering has become a prominent trend, significantly advancing the diagnosis and treatment of tumors.^73^, ^74^ The successful application of AI in medical imaging has enabled AI‐based cancer imaging analysis technologies to address complex clinical needs, such as predicting the cancer prognosis, predicting treatment responses, distinguishing between benign and malignant lesions, identifying abnormal tumor responses, and predicting mutations and molecular features.^75^, ^76^ In the field of LCBM, AI and imaging omics have proven effective in distinguishing BM from different primary tumors.^77^ Gao et al.^78^ developed a deep learning model for the automatic identification and classification of 18 types of brain tumors. This model, using T1‐weighted gradient‐echo MRI scans, can detect nearly all BM that are 6 mm or larger with a low false positive rate.^79^ A systematic review and meta‐analysis conducted by Wang et al.,^80^ which included 42 studies demonstrated that deep learning models, particularly U‐Net and its variants, excel in segmentation accuracy.^81^ Yun applied these deep learning models in prospective studies, noting improvements in detection performance, though challenges remain for small BM. Enhancing the detection sensitivity for small metastatic lesions, may require larger training datasets and refined network designs.^79^, ^82^, ^83^

Deep learning also plays a crucial role in identifying the primary sources of BM. Jiao's retrospective study, which included BM patients from various tumors (100 SCLC patients, 125 NSCLC patients, 116 BC patients, and 108 gastrointestinal cancer patients), utilized a three‐dimensional residual network (3D‐ResNet) to identify the tumor origin. The model could distinguish between LC and non‐LC, SCLC, and NSCLC, BC and gastrointestinal cancer using MRI sequences like T1WI, DWI, and CE‐T1WI. However, combining MRI sequences such as CE‐T1WI + T2WI + DWI improved differentiation between BC and gastrointestinal cancer. but did not accurately distinguish LC from non‐LC.^84^

To predict the incidence of LCBM, enable early detection and accurate classification, clinical, pathological, imaging, and other omics data are integrated. A deep learning algorithm has achieved an 87% accuracy rate in predicting BM development, significantly outperforming the average accuracy of four pathologists (57.3%).^85^ Jeong et al.^86^ found that sensitivity for detecting high‐risk patients was 95%.^87^ Targeted therapy for LCBM often relies on pathological information from the LC. However, the discrepancies between BM and primary LC histology have led to the use of multitask deep learning networks to predict molecular classifications such as epidermal growth factor receptor (EGFR) wild‐type and mutant types.^88^, ^89^ Additionally, deep learning models for EGFR 19Del/21L858R mutations and wild type have achieved AUC values above 0.97, though precautions are needed to address potential issues such as deceptive strategies and overfitting in AI.^86^

Combining clinical information with MRI‐based deep learning facilitates the segmentation of BM gross tumor volume and predicts RT efficacy.^88^, ^90^, ^91^, ^92^, ^93^, ^94^ Deep learning radiomics and EGFR status are used to predict survival after SRS for LCBM. Combining deep learning with imaging data before and after whole‐brain radiation therapy (WBRT), Rammohan et al.^95^ demonstrated that the aging rate of the brain and changes in brain substructure accelerated after WBRT, which are associated with neurocognitive function and could guide clinical treatment. Beyond RT, AI is also employed to predict the efficacy of targeted therapy, immunotherapy, and other treatments.^96^, ^97^, ^98^ Recent advancements include the use of deep learning for intraoperative brain tumor identification and near‐real‐time diagnosis using simulated Raman histology and deep neural networks.^99^, ^100^ Radiogenomics, which examines the relationship between genomics and imaging phenotypes, has been instrumental in addressing tumor heterogeneity and predicting immune responses and progression.^101^ However, unveiling the “black box” of radiomics imaging will contribute to the development of precision medicine research.^102^

In BCBM, AI primarily focuses on predicting the occurrence of BM, identifying the primary lesions, and predicting molecular subtypes. A multivariate logistic regression model using clinical variables at diagnosis achieved AUC values of 0.95, 0.94, 0.77, and 0.61 for BC, melanoma, and NSCLC/SCLC, respectively.^2^, ^103^ Deep learning models have been used to identify the primary lesions of BM by analyzing anatomical distribution differences.^44^ HER2 status has been predicted with high accuracy based on relative cerebral blood volume, achieving a model accuracy of 0.98.^104^ Preoperative brain MRI combined with deep learning has predicted ER, PR, and HER2 status with accuracies of 0.89, 0.88, and 0.87, respectively.^105^, ^106^ However, Strotzer et al.^107^ noted limitations in predicting BM histology based on MRI. Additionally, AI also aids in forecasting prognosis and guiding treatment for BM patients, with the XGBoost model predicting the 6‐month to 3‐year prognosis for BC patients, achieving AUC values exceeding 0.8.^108^ Pandey et al.^109^ developed a deep learning framework to optimize SRS dose planning for BCBM patients using multiparametric MRI images.

Despite promising prospects, machine learning and radiomics may not always be reliable. Deep learning exhibits limitations such as deceptive predictions and overfitting.^110^ The auxiliary role of AI is powerful, but whether it can be independently applied to the diagnosis and treatment of BM remains an open question.^86^ Future development will focus on creating more practical and accurate algorithms, adopting refined imaging techniques, and continuing the integration of medicine and engineering.^80^

In general, diagnosing BM remains relatively straightforward, whether during the initial treatment or throughout the course of the primary tumor. In the era of AI, machine learning is primarily used to predict the occurrence and prognosis of BM, locate primary lesions, and assess treatment responses. However, prediction models’ accuracy can be limited by small sample sizes and noninnovative algorithms. Additionally, the lack of explainability in AI restricts their further application.

## 3 TREATMENTS OF METASTATIC BRAIN TUMORS

### 3.1 LCBM

The treatment of LCBM mainly include surgery, RT, chemotherapy, targeted therapy, and immunotherapy.^29^, ^111^ Here, we briefly describe the treatment strategies.

#### 3.1.1 Surgical treatment

Surgery is often necessary for BM, particularly when they cause significant intracranial hypertension.^112^ Neurosurgeons evaluate the need for surgery, which can quickly relieve symptoms, potentially achieve local cure by completely removing the tumor and provide tumor tissue for pathological diagnosis.^113^ Postoperative adjuvant therapy, specifically RT combined with immunotherapy, have shown improved OS compared with chemoradiotherapy (23.0 vs. 11.8 months).^114^ However, patients without surgical indications typically receive nonsurgical treatments following supportive care.

#### 3.1.2 Targeted therapy

For asymptomatic BM, targeted therapy is a primary treatment based on driver gene status.^115^, ^116^ The prognosis of patients with EGFR mutations (e.g., exon 19 deletion and exon 21 mutation) and ALK rearrangement have improved with targeted therapy.^116^, ^117^ Targeted therapy has reduced the need for local treatment for BM.^118^, ^119^ Recent advancements also benefit patients with rare mutations like KRAS G12 mutations, MET ex14 and EGFR 20ins.^120^, ^121^ Patients unable to receive targeted therapy may consider further treatments such as chemotherapy, immunotherapy, RT, antiangiogenesis. Additionally, for patients receiving targeted therapy, short‐term intracranial progression is mainly attributed to residual lesions, which may be addressed with local treatments like surgery, SRS, or WBRT.^122^ Additionally, issues such as drug resistance after targeted therapy, the lack of available drugs for the target, or poor efficacy can also arise.^123^

#### 3.1.3 Radiotherapy

RT is a crucial local treatment for LCBM.^124^ It is primarily categorized into SRS and WBRT. SRS is typically utilized for oligo BM (usually fewer than four lesions), whereas WBRT is preferred for more extensive metastases. Both SRS and WBRT have limitations such as radiation‐induced brain necrosis, neurocognitive impairment, and posttreatment progression.^125^, ^126^ However, given that SCLC tends to progress and metastasize widely, current clinical guidelines recommend routine MRI or prophylactic cranial irradiation.^127^

In recent years, there has been increasing scrutiny regarding the application of SRS for multiple BM, with the introduction of hippocampal avoidance radiotherapy (HA–WBRT) helping to mitigate damage to neurocognitive function.^128^ The therapeutic benefits of SRS have prompted researchers to explore its expanded indications. However, Bodensohn et al.’s^129^ study confirmed that applying SRS to patients with 4–10 BM lesions did not effectively improve OS. Moreover, there is insufficient high‐quality evidence regarding the separation and combination of SRS and WBRT. Compared with WBRT combined with SRS, WBRT with simultaneous integrated boost did not significantly alter median OS and objective response rate (ORR) but did extend median intracranial PFS (iPFS).^130^ The debate between SRS and WBRT remains ongoing. Compared with SRS, WBRT with simultaneous integrated boost did not significantly prolong OS, but the median iPFS was longer.^131^ Ni et al.^132^ retrospectively analyzed that WBRT plus focal radiation boost resulted in prolonged OS and iPFS compared with WBRT or SRS alone.

RT represents an important local treatment that can prolong the survival of patients.^133^, ^134^ Whether prophylactic brain irradiation can replace WBRT and reduce neurocognitive impairment remains unknown. Current research is also investigating the use of prophylactic brain irradiation in patients with stage III or pathologically node‐positive NSCLC, as exemplified by studies such as NCT02448992. As an important component of BM treatment, local therapies like physical therapies such as TTFields (NCT02831959) and focused ultrasound (NCT05317858) have also garnered attention. The METIS study, which is announced at the 2024 ASCO meeting, focuses on the combined application of SRS and TTFields. METIS study indicated that TTFields following SRS can significantly delay median intracranial survival time (SRS + TTFields vs. SRS + best supportive care: 21.9 vs. 11.3 months). Furthermore, patients treated with TTFields tolerated the therapy well, experiencing significant improvements in quality of life and PFS.

#### 3.1.4 Chemotherapy

Despite the challenges with penetrating the BBB, chemotherapy remains vital for treating LCBM.^135^ Agents like pemetrexed and temozolomide are pivotal, and antiangiogenic drugs such as bevacizumab have shown superior ORR and disease control rate (DCR) for intracranial lesions compared with extracranial lesions, without increasing the risk of bleeding in BM patients.^136^, ^137^, ^138^ Recent trials support combining platinum–pemetrexed with osimertinib can effectively manage the progression of EGFR + LCBM (NCT04035486), positioning it as a viable first‐line treatment option. Moreover, phase III clinical trial (NCT01951469) support gefitinib combined with chemotherapy as a first‐line treatment for untreated EGFR + LCBM.^139^

#### 3.1.5 Immunotherapy

Immunotherapy has significantly advanced treatment landscape of LC in recent years, leveraging the immune system to target tumor cells.^140^, ^141^ Immune checkpoint inhibitors (ICIs) have particularly revolutionized the management of nononcogene‐driven NSCLC.^142^ However, comprehensive treatment remains pivotal for managing LCBM. The multicenter ESCKEYP GFPC study demonstrated that there was no significant difference in ICI response rates or PFS between patients with and without BM at baseline, highlighting the robust therapeutic efficacy of ICIs.^143^, ^144^

Optimizing therapeutic effect through combinations of immunotherapy, chemotherapy, and RT remains ongoing. Meta‐analyses and SEER database analysis by Abdulhaleem et al.^145^ have confirmed that immunotherapy extends OS.^145^, ^146^ Furthermore, combining dual ICIs or single ICI with chemotherapy has shown superior OS extension, along with higher incidence of treatment‐related adverse events.^147^ Prospective studies have validated that dual ICI regimens, such as nivolumab plus ipilimumab, significantly prolong 5‐year systemic and intracranial PFS following immunotherapy.^148^ In the context of local BM treatment, numerous studies support RT is pivotal in overcoming the BBB and enhancing immunotherapy efficacy. Compared with WBRT, simultaneous SRS with ICI demonstrates enhanced effectiveness.^149^, ^150^ Meta‐analyses by Yu et al.^151^ corroborate that synchronized ICI and RT achieve optimal outcomes without significantly increasing adverse events. However, Li et al.’s^152^ retrospective analysis indicates that ICI may elevate the risk of radiation necrosis, particularly within 3 months post‐RT. Augmenting efficacy through the addition of G‐CSF in radioimmunoassay further enhances treatment outcomes.^153^, ^154^ Another strategy for advanced NSCLC treatment involves dual ICI combinations (anti‐PD1/anti‐PD‐L1 and anti‐CTLA4), which synergistically optimize cell‐mediated immune responses against tumor cells.^154^ Phase I/II clinical trials have validated the safety of dual ICIs (nivolumab and ipilimumab) in conjunction with SRS for LCBM.

The role of immunotherapy in treating BM from SCLC remains a subject of ongoing exploration and debate.^155^ Retrospective studies have yielded conflicting results regarding the efficacy of combining RT with ICI in SCLC. Some findings suggest that RT combined with ICI does not confer significant survival or local control benefits for SCLC BM.^156^ For instance, CASPIAN, IMpower133, and ASTRUM‐005 did not demonstrate a clear OS advantage in SCLC patients with BM.^157^, ^158^, ^159^, ^160^ Specifically, meta‐analyses by Zhou et al.^161^ indicated that the addition of ICI to chemotherapy did not improve OS compared with chemotherapy alone (HR = 1.23), although it did prolong PFS (HR = 0.81). The ORR were similar between the two treatment groups (RR = 1.04).^161^ ASTRUM‐005 similarly showed no significant OS benefit irrespective of BM status at baseline (HR = 0.62 vs. 0.61).^160^ Retrospective studies have also produced conflicting data on the combination of RT and ICI for SCLC BM. While some suggest no survival benefit or increased neurotoxicity, others indicate potential survival improvements, especially when WBRT precedes ICI treatment.^156^, ^162^ Additionally, Lu et al.^163^ found that ICI therapy did not delay brain progression or reduce the risk of intracranial metastasis in SCLC. Despite these challenges, the combination of chemoradiotherapy and immunotherapy remains a viable treatment option for SCLC.^164^ Further prospective studies and clinical trials are needed to clarify the optimal treatment strategies and patient selection criteria for immunotherapy in SCLC BM.^142^

The landscape of clinical trials focusing on LCBM reflects ongoing efforts to refine diagnosis and treatment approaches, though challenges persist, particularly in addressing active or untreated BM. Currently, approximately 250 clinical studies registered on ClinicalTrials.gov are dedicated to exploring the treatments of LCBM. These trials encompass both diagnostic innovations, such as new PET–CT methodologies (NCT00253461, NCT05452005, NCT00040560, NCT04752267, NCT00445965, etc.), and therapeutic strategies centered around targeted therapy, RT, chemotherapy, and physical treatments. The majority of these trials fall within the phase I–II, which primarily aims to assess safety, dosage, and initial efficacy. In contrast, phase III–IV trials that provide robust evidence for clinical applications are relatively less common (Table 1). Phase III trials predominantly evaluate local treatments and targeted therapies, while immunotherapy trials are comparatively rare. Some studies are also investigating innovative approaches like vaccine therapies involving dendritic cells and macrophages, reflecting a broader therapeutic strategy (NCT01782287). The identification of specific targets, such as HER3, has further spurred the development of novel ADCs. Phase III trial HERTHENA‐Lung01 demonstrated ADC's efficacy in EGFR‐resistant or postchemoimmunotherapy scenarios.^165^ Despite these advancements, the complex anatomical considerations and unique microenvironment of BM pose significant treatment challenges. Recent developments in single‐cell/spatial transcriptomics have shed light on the underlying mechanisms and microenvironmental nuances of BM. However, treatment strategies directly targeting these insights remain limited. Moving forward, bridging the gap between retrospective findings and prospective clinical trials will be crucial.

**TABLE 1:** Phase III clinical trials for BM from 2021 to 2024.

Therapy	Author	Clinical Trial ID	Year	Phase	Inclusion Patients	Interventions	Patients, *N*	Results	AEs	Conclusions	References
**TKI alone**											
TKI	Pérol, Maurice et al.	LIBRETTO‐431 (NCT04194944)	2024	III	RET fusion+ NSCLC (CNS analysis)	Selpercatinib vs. platinum/pemetrexed ± pembrolizumab	42	Among patients with BM at baseline: selpercatinib group: 12‐month intracranial CR of CNS progression: 25.7%; CR rate: 42.9%; median time to intracranial response: 1.4m; median intracranial DOR: not reached; 12‐month intracranial DOR rate: 80.7%; median intracranial PFS: not reached; 12‐month intracranial PFS rate: 63.9%; Control group: 12‐month intracranial CR of CNS progression: 33.3%; CR rate: 33.3%; median time to intracranial response: 2.2m; median intracranial DOR: not reached; 12‐month intracranial DOR rate: 75.8%	NA	Selpercatinib effectively treats existing CNS disease and prevents or delays the formation of new CNS metastases.	^166^
TKI	Yang, Yunpeng et al.	NCT04009317	2023	III	ALK+ NSCLC with BM	Envonalkib vs. crizotinib	88	Patients with baseline intracranial target lesions: envonalkib group: CNS‐ORR: 78.95%; DOR: 25.82m; CNS‐TTP: 26.68m; crizotinib group: CNS‐ORR: 23.81%; DOR: 7.39m; CNS‐TTP: 6.34m; patients with BM at baseline: envonalkib group: CNS‐TTP: 30.32m; crizotinib group: CNS‐TTP: 8.28m	Most common TEAEs in the envonalkib group: diarrhea, vomiting, elevated alanine transaminase, nausea, and elevated AST	Envonalkib significantly improved PFS and delayed BM progression in advanced ALK + NSCLC.	^116^
TKI	Solomon, Benjamin J et al.	CROWN (NCT03052608)	2023	III	ALK+ advanced NSCLC	Lorlatinib vs. crizotinib	76	Patients with measurable and nonmeasurable baseline BM: lorlatinib group: intracranial ORR: 65%; median intracranial DOR: NR; mTTI: not reached; crizotinib group: intracranial ORR: 18%; median intracranial DOR: 9.4m; median time to intracranial progression: 7.3m; patients with measurable baseline BM: lorlatinib group: intracranial ORR: 83%; median intracranial DOR: Not reached; crizotinib group: intracranial ORR: 23%; median intracranial DOR: 10.2m	Grade 3–4 AEs occurred in lorlatinib and crizotinib: 76 and 57%. No new safety signals	Durable benefit of lorlatinib over crizotinib in patients with treatment‐naive, ALK + NSCLC and support the use of first‐line lorlatinib in patients with and without baseline BM.	^167^
TKI	Ahn, Myung J et al	ALTA‐1L (NCT02737501)	2022	III	ALK inhibitor‐naive ALK+ NSCLC (Asian vs. non‐Asian patients)	Brigatinib vs. crizotinib	96	In Asian patients: brigatinib group: intracranial ORR: 62%; crizotinib group: intracranial ORR: 33% In non‐Asian patients: brigatinib group: intracranial ORR: 69%; crizotinib group: intracranial ORR: 3%	Most common TEAEs: gastrointestinal events, increased blood creatine phosphokinase (CPK), cough, increased aminotransferases, and peripheral edema	Efficacy with brigatinib was consistently better than with crizotinib in Asian and non‐Asian patients with locally advanced or metastatic ALK inhibitor‐naive ALK− + NSCLC. There were no clinically notable differences in overall safety in Asian vs. non‐Asian patients	^168^
TKI	Solomon, Benjamin J et al.	CROWN (NCT03052608)	2022	III	Locally advanced or metastatic ALK+ NSCLC (post hoc analysis)	Lorlatinib vs. crizotinib	296	Patients with BM at baseline: lorlatinib group: 12‐month PFS rates: 78%; 12‐month cumulative incidence of CNS progression: 7%; CRIZOTINIB group: 12‐month PFS rates: 22%; 12‐month cumulative incidence of CNS progression: 72%	35% of patients had CNS AEs with lorlatinib, most of grade 1 severity.	First‐line lorlatinib improved PFS and reduced CNS progression versus crizotinib in patients with advanced ALK + NSCLC with or without BM at baseline. Half of all CNS AEs resolved without intervention or with lorlatinib dose modification.	^169^
TKI	D Ross Camidge et al.	ALTA‐1L (NCT02737501)	2021	III	NSCLC with BM not received ALK‐targeted therapy	Brigatinib vs. crizotinib	81	Brigatinib group: mDOR: 27.9m; 3‐year PFS: 31%; intracranial ORR: 31/47; crizotinib group: mDOR: 9.2m; 3‐year PFS: 9%; intracranial ORR: 7/49	NA	Survival benefit with brigatinib in patients with BM warrants future study.	^170^
TKI	Leora Horn et al.	NCT02767804	2021	III	ALK+ NSCLC with asymptomatic BM	Ensartinib vs. crizotinib	104	mPFS: ensartinib group: 11.8m; crizotinib group: 7.5m	NA	Ensartinib showed superior efficacy to crizotinib in intracranial disease.	^171^
TKI	Tu, Hai‐Yan et al.	NCT01953913	2022	III	EGFRm NSCLC (focus on patients enrolled in China)	Afatinib	84	Patients with BM from China: time to symptomatic progression: 11.0m; PFS: 9.2m	Most common AE and grade ≥3 TRAEs: diarrhea, rash/acne, and stomatitis	Tolerability‐guided afatinib dose reduction allowed patients to remain on treatment and continue to experience clinical benefit.	^172^
TKI	Filippo de Marinis et al.	NCT01853826	2021	III	EGFR TKI‐naïve patients with brain metastatic EGFRm NSCLC	Afatinib	83	Median time to symptomatic progression: 13.7m; mPFS: 10.1m; mDOR: 11.1m; median disease control rate: 11.6m	Most common any grade TRAEs: diarrhea, rash, paronychia, mucosal inflammation, dry skin, stomatitis, skin fissures, nausea, dermatitis acneiform, and conjunctivitis	Afatinib was well tolerated with no new safety signals and demonstrated promising efficacy in patients with EGFRm NSCLC.	^173^
**TKI combination**											
TKI + TKI	Zhou, Hua‐Qiang et al.	FL‐ALTER (NCT04028778)	2024	III	Untreated, EGFRm, advanced NSCLC	Gefitinib + anlotinib vs. gefitinib + placebo	99	Among patients with BM: gefitinib + anlotinib group: mPFS: 13.8m; a 53% reduction in the risk of progression; gefitinib + placebo group: mPFS: 8.3m	Incidence of grade 3 or higher TRAE of gefitinib + anlotinib:49.7%; gefitinib + placebo:31.0%	Patients with BM and those harboring EGFR amplification or high tumor mutation load gained significant more benefits in PFS from gefitinib + anlotinib.	^174^
TKI + chemo	Jänne, Pasi A et al.	FLAURA2 (NCT04035486)	2024	III	EGFRm advanced NSCLC with BM (CNS efficacy analysis)	Osimertinib + platinum‐pemetrexed (combination) vs. osimertinib monotherapy	222	Combination arm: miPFS: 30.2m; estimated probability of observing a CNS progression event at 24 months: 9%; CNS ORRs: 73%; intracranial CR: 59%; median time to response: 11.8 weeks; median intracranial DOR: not reached; median best percentage change from baseline in CNS target lesion size: −94%. Monotherapy arm: miPFS: 27.6m; estimated probability of observing an intracranial progression event at 24 months: 23%; intracranial ORRs: 69%; intracranial CR: 43%; median time to response: 8.4 weeks; median intracranial DOR: 26.2m; median best percentage change from baseline in CNS target lesion size: −61%	AE rates were similar between the CNS full analysis set (cFAS) and the overall FLAURA2 study population	Osimertinib + platinum‐pemetrexed demonstrated improved CNS efficacy compared with osimertinib monotherapy, including delaying CNS progression, irrespective of baseline CNS metastasis status.	^175^
TKI + chemo	Hou, Xue et al.	GAP‐BRAIN (NCT01951469)	2023	III	Untreated EGFRm‐NSCLC with BMs	Gefitinib + chemotherapy vs. gefitinib	167	Gefitinib + chemotherapy group: miPFS: 15.6m; mPFS: 16.3m; intracranial ORR: 85.0%; overall ORR: 80.0%; mOS: 35.0m; gefitinib group: miPFS: 9.1m; mPFS: 9.5m; intracranial ORR: 63.0%; overall ORR:64.2%; mOS: 28.9m	Most common grade 3 or worse AEs: ALT increase. Grade 3 or worse AEs were more common with gefitinib + chemotherapy.	Gefitinib + chemotherapy significantly improved intracranial PFS, PFS, and OS compared with gefitinib alone in patients with untreated EGFR‐mutant NSCLC BM and could be an optional first‐line treatment for these patients.	^139^
TKI + anti‐VEFG	Qing Zhou et al.	ARTEMIS‐CTONG1509 (NCT02759614)	2021	III	Untreated NSCLC patients with BM	Bevacizumab + erlotinib vs. erlotinib	91	Bevacizumab + erlotinib arm: mPFS: 17.9m; mOS: 31.6m; erlotinib arm: mPFS: 11.1m; mOS: 26.8m	NA	Bevacizumab + erlotinib significantly improved PFS in EGFRm NSCLC patients with untreated BM.	^138^
**Immunotherapy combination**											
ICI + chemo + ADC	Rudin, Charles M et al.	SKYSCRAPER‐02 (NCT04256421)	2024	III	Untreated extensive‐stage SCLC	Tiragolumab + atezolizumab and carboplatin and etoposide (CE) vs. placebo + atezolizumab and CE	93	Tiragolumab + atezolizumab group: mOS: 11.7m; mOS of patients with treated BM: 12.4m; mOS of patients with untreated BM: 11.7m; control group: mOS: 10.8 m; mOS of patients with treated BM: 15.7m; mOS of patients with untreated BM: 10.2m	Most common grade 3/4 TRAEs: anemia and neutropenia. Most common severe AEs: febrile neutropenia and pneumonia. No new safety signals.	Tiragolumab did not provide additional benefit over atezolizumab and CE in untreated ES‐SCLC. The combination was well tolerated with no new safety signals.	^176^
ICI + ICI/ICI + chemo	Reck, Martin et al.	CheckMate 227 (NCT02477826)	2023	III	Metastatic NSCLC with baseline BM (post hoc exploratory systemic)	PD‐L1 greater than or equal to 1%: nivolumab + ipilimumab/nivolumab/chemotherapy. Tumor PD‐L1 less than 1%: nivolumab + ipilimumab/nivolumab + chemotherapy/chemotherapy	202	Nivolumab + ipilimumab group: mOS: 17.4m; 5‐year OS rates: 20%; 5‐year systemic and intracranial PFS rates: 12 and 16%; incidence of developing new brain lesions: 4%; systemic ORR: 32%; mDOR: 24.9m; chemotherapy group: mOS: 13.7m; 5‐year OS rates: 6%; 5‐year systemic and intracranial PFS rates: 0% and 6%; incidence of developing new brain lesions: 20%; systemic ORR: 26%; mDOR: 8.4m	Most common any‐grade neurologic TRAEs: headache, paresthesia, taste disorder, and dysgeusia. No new safety signals	Nivolumab + ipilimumab continued to provide a long‐term, durable survival benefit in patients with or without BM. Intracranial efficacy outcomes favored nivolumab + ipilimumab versus chemotherapy.	^148^
ICI + ICI	Ready, Neal E et al.	CheckMate817 (NCT02869789)	2023	III	Metastatic NSCLC (special cohort analysis)	Nivolumab + ipilimumab	49	mOS: 12.8m; 3‐year OS rate: 21%; mPFS: 2.8m; 3‐year PFS rate: 14.2%; ORR: 32.7%; mDOR: 12.6m; 39% of responders had an ongoing response at 3 years	Most common grade 3–4 immune‐mediated AEs: diarrhea/colitis, hepatitis, and pneumonitis. Most common grade 3–4 treatment‐related select AEs: gastrointestinal and pulmonary events	Special populations of cohort A1 including patients with ECOG PS 2 or ECOG PS 0–1 with untreated BM had manageable treatment‐related toxicity and clinically meaningful 3‐year OS rate.	^177^
**Others**											
PD‐1 + VEGF + chemo	Wenfeng Fang et al.	NCT05184712	2024	III	Relapsed advanced or metastatic EGFRm NSCLC	Ivonescimab + pemetrexed and carboplatin vs. placebo + pemetrexed and carboplatin	72	Among patients with BM at baseline: ivonescimab + pemetrexed + carboplatin group: mPFS: 5.75m; a 60% reduction in the risk of progression; placebo + pemetrexed + carboplatin group: mPFS: 4.14m	Most common grade 3 or higher TRAE: chemotherapy related.	Ivonescimab + chemotherapy significantly improved PFS with tolerable safety profile in TKI‐treated NSCLC.	^178^
**RT**											
**RT alone**											
RT	Zeng, Ming et al.	HYBRID (NCT02882984)	2024	III	EGFRm NSCLC with BM	WBRT vs. SRS	85	WBRT group: intracranial progression at 18 months: 9.5%; miPFS: 21.4m SRS group: intracranial progression at 18 months: 10.2%; miPFS: 22.3m	NA	The SRS arm experienced higher overall survival and cognitive preservation. Although this phase III trial was underpowered, there was no evidence that SRS yielded outcome detriments compared with WBRT for EGFRm NSCLC BMs.	^179^
**RT combination**											
RT + TKI	Zhenzhou Yang et al.	NCT01887795	2021	III	NSCLC with multiple BM	WBRT vs. WBRT + erlotinib	224	WBRT arm: miPFS: 9.1m; mPFS: 5.3m; mOS: 12.9m; WBRT + erlotinib arm: miPFS: 11.2m; mPFS: 4.0m; mOS: 10.0m	Most common AE: drug‐related acneiform rash. Significance differences between the two arms: acneiform rash, dry skin, increased AST/ALT, increased bilirubin, paresthesia, and cough	Concurrent erlotinib with WBRT did not improve iPFS and excessive CF detriment either in the intent‐to‐treat (ITT) population or in EGFR‐mutant patients compared with WBRT alone.	^180^
**Breast cancer**											
Antibody–drug conjugate (ADC)	Hurvitz, S A et al.	DESTINY‐Breast03 (NCT03529110)	2024	III	HER2+ metastatic BC previously treated with trastuzumab and a taxane	T‐DXd vs. T‐DM1	82	T‐DXd group: mPFS: 15m; ORR 67.4%; intracranial ORR 65.7%; T‐DM1 group: mPFS: 3.0 m; ORR 20.5%; intracranial ORR 35.3%	NA	Patients with HER2+ metastatic BC whose disease progressed after trastuzumab and a taxane achieved a substantial benefit from treatment with T‐DXd compared with T‐DM1, including those with baseline BMs.	^181^
Chemotherapy (Chemo)	Tripathy, Debu et al.	ATTAIN (NCT02915744)	2022	III	Metastatic BC with BM	Etirinotecan pegol vs. chemotherapy	178	Etirinotecan pegol group: mOS: 7.8m; mPFS for CNS metastasis: 3.9m; chemotherapy group: mOS: 7.5m; mPFS for CNS metastasis: 3.3m	Most common treatment‐related AEs: diarrhea, nausea, fatigue, vomiting, decreased appetite, asthenia, neutropenia, anemia, abdominal pain, constipation, headache, decreased weight, alopecia, decreased neutrophil count, peripheral neuropathy. Comparable safety profiles between the groups	No statistically significant difference in outcomes between treatment with etirinotecan pegol and chemotherapy in patients with BM	^182^
TKI + chemo	Dai, Ming Shen et al.	NALA (NCT01808573)	2021	III	HER2+ metastatic BC (Asian subgroup in the NALA study)	Neratinib + capecitabine (N+C) vs. lapatinib + capecitabine (L+C)	43	N+C group: interventions for CNS disease: 16(15.4%); overall cumulative incidence of intervention for CNS disease: 27.9%; L+C group: interventions for CNS disease: 27(27.6%); overall cumulative incidence of intervention for CNS disease: 33.8%	Most frequent TEAEs: diarrhea and palmar‐plantar erythrodysesthesia; comparable incidences of grade 3/4 TEAEs and TEAEs leading to treatment discontinuation. No new safety signals	Asian patients with HER2+ metastatic BC, who had received ≥ 2 HER2‐directed regimens, may also benefit from N+C.	^183^
TKI + chemo	Hurvitz, Sara A et al.	NALA (NCT01808573)	2021	III	HER2+ metastatic BC (patients with CNS metastases at baseline from the NALA trial)	Neratinib + capecitabine (N+C) vs. lapatinib + capecitabine (L+C)	101	N+C group: mean PFS through 24 month: 7.8m; mPFS: 5.6m; mean OS through 48 months: 16.4m; mOS: 13.9m; cumulative incidence of interventions for CNS disease at 6 months and at 12 months: 15.7 and 25.5%; cumulative incidence of progressive CNS disease at 12 months: 26.2%; miPFS:12.4 m; intracranial ORR: 26.3%; L+C group: mean PFS through 24 month: 5.5m; mPFS: 4.3m; mean OS through 48 months: 15.4m; mOS: 12.4m; cumulative incidence of interventions for CNS disease at 6 months and 12 months: 24.0% and 36%; cumulative incidence of progressive CNS disease at 12 months: 41.6%; miPFS: 8.3m; confirmed intracranial ORR: 15.4%	Most common TEAEs of any grade: diarrhea, nausea, vomiting, and palmar‐plantar erythrodysesthesia syndrome; Common CNS AEs (grade 1–4): headache, dizziness, hemiparesis, seizure, and gait disturbance. No new safety signals	The combination of neratinib and capecitabine was associated with improved PFS and CNS outcomes compared with lapatinib and capecitabine in patients with CNS metastases from HER2+ metastatic BC.	^184^

Abbreviations: AE, adverse events; ALK, anaplastic lymphoma kinase; CNS, central nervous system; CR, complete response; DOR, duration of response; DBF, distant brain failure; EGFRm, epidermal growth factor receptor mutated; FF, freedom from; iPFS, intracranial progression free survival; HER2, human epidermal growth factor receptor 2; OS, overall survival; ORR, objective response rate; OR, overall response; RT, radiotherapy; SRS, stereotactic radiosurgery; PFS, progression free survival; WBRT, whole brain radiotherapy; T‐DXd, trastuzumab deruxtecan; T‐DM1, trastuzumab emtansine; TEAE, treatment emergent adverse events; TMZ, temozolomide; TRAE, treatment‐related adverse event; TKI, tyrosine kinase; TTP,s time to progression.

BM is mainly treated with comprehensive approaches. After utilizing targeted therapy, immunotherapy, RT, and other modalities, the prognosis of LCBM has significantly improved. Local treatments primarily include surgery and RT, while systemic treatments such as targeted therapy and immunotherapy have largely supplanted chemotherapy. In addition to considering the dose and fractionation of RT, the focus has shifted to employing HA–WBRT and SRS to mitigate neurocognitive dysfunction. The integration of targeted therapy, immunotherapy, and RT is a prominent topic in BM. Factors such as optimal dosing, treatment sequencing, and timing all influence efficacy.^185^ The latest studies have highlighted the therapeutic potential of TTFields and have also directed attention toward physical therapy.

### 3.2 BCBM

#### 3.2.1 Surgical treatment

Surgery significantly reduces intracranial pressure in patients with multiple BMs while also allowing for tumor tissue acquisition.^186^ Labeling BCBM with 5‐aminolevulinic acid enhances surgical resection and prolongs survival.^187^ However, perioperative stress and inflammatory signals may promote tumor metastasis and impact surgical effectiveness. Hanalis‐Miller et al.^188^ demonstrated that personalized psychological interventions for perioperative patients can reduce the expression of tumor metastasis‐related molecules. Combining systemic therapy with local therapy remains crucial for effective treatment. Hijazi et al.^189^ analyzed data from 9005 BCBM patients using the National Cancer Database and found that patients who received only local treatment without systemic therapy had more than double the risk of death compared with those who received systemic treatment.

#### 3.2.2 Systemic therapy

For BCBM patients, determining the molecular subtype is essential for guiding treatments. HER2+ BC and TNBC are known to frequently metastasize to the brain. Recent studies have highlighted the importance of identifying patients with low or no HER2 expression. Onder and Karacin et al.^190^ retrospectively analyzed 201 BC patients and found that the median BM‐free survival was 43.7 months for patients with low HER2 expression and 30.1 months for those with no HER2 expression. Interestingly, the survival period after the occurrence of BM was similar in both groups.^190^ This underscores the importance of accurate molecular subtypes identification for tailoring treatment strategies for BCBM.

Anti‐HER2 therapy plays a crucial role in managing HER2+ BCBM patients. After trastuzumab deruxtecan treatment, the mPFS for patients with active BMs and leptomeningeal metastases was 13.2 months and 17.5 months, respectively, while the survival for patients with stable BM had not yet reached 20 months of follow‐up.^191^, ^192^ Meta‐analysis further supports the efficacy of trastuzumab deruxtecan, showing an intracranial ORR of 61%. Specifically, the ORR for patients with stable BM was 68%, while it was 60% for patients with active BM.^193^, ^194^ The phase III DESTINY‐Breast03 trial also demonstrated that trastuzumab deruxtecan significantly improved PFS for HER2+ BCBM patients.^195^

Tyrosine kinase inhibitors (TKIs) are effective targeted therapies for HER2+ BC, as they inhibit the tyrosine kinase activity of both the EGFR and HER2. TKIs, including neratinib, lapatinib, and pyrotinib, have demonstrated the ability to prolong the prognosis of BMs. Recent phase II clinical trials highlight the significant efficacy of neratinib, showing its effectiveness in treating newly diagnosed or previously treated BCBM patients.^196^ Similarly, pyrotinib combined with trastuzumab has been shown to extend the mPFS of HER2+ BCBM patients to 17.9 months.^197^

Combining TKIs with chemotherapy significantly improves the intracranial ORR. Wang et al.^11^ demonstrated that the combination of pyrotinib and capecitabine in BCBM patients (who had not received/had received prior RT, or who had progressed after RT) achieved intracranial ORRs of 72.73, 55, and 42.86%, respectively. Prospective studies have shown that the intracranial ORR of pyrotinib combined with capecitabine in HER2+ BCBM patients who had not received treatment was 74.6%, while the intracranial ORR of patients who had previously received trastuzumab was 42.1%.^198^ However, Mikaeili Namini et al.^199^ showed that there was no significant difference in the intracranial ORR of HER2+ BCBM patients with pyrotinib combined with nab‐paclitaxel, capecitabine, or vinorelbine, although the peripheral ORR was relatively better with nab‐paclitaxel, suggesting it may be a preferred option.

Clinical trials exploring multidrug combinations are currently underway. In BCBM, systemic treatment with TKIs such as tucatinib, lapatinib, and pyrotinib has been shown to prolong PFS.^200^ A phase II prospective study by Chen et al.^201^ demonstrated that a combination of palbociclib, trastuzumab, pyrotinib, and fulvestrant may offer a new treatment option for HR+ HER2+ BCBM patients. Additionally, tucatinib combined with trastuzumab and capecitabine may effectively treat HER2+ BCBM patients.^202^ Huo et al.^203^ demonstrated through a network meta‐analysis that the ORR of the trastuzumab deruxtecan and pyrotinib combined with capecitabine regimen was particularly significant (ORR 73.33%).

#### 3.2.3 Radiotherapy

RT remains a crucial local treatment for BCBM, commonly including SRS and WBRT. The most frequently used WBRT regimen is 30 Gy in 10 fractions. Among BM patients from various primary tumors (BC, NSCLC, SCLC, or melanoma) who received WBRT, those with BC had the longest survival time, with a mOS of approximately 7.7 months.^204^ Further research indicates that combining WBRT with simultaneous integrated boost enhance treatment efficacy for BCBM.^205^ However, similar to LCBM patients, WBRT can lead to cognitive and neurological deficits.^206^ A retrospective analysis of 873 BCBM patients at MD Anderson Cancer Center found that SRS, surgery, or SRS followed by WBRT had comparable OS and local control.^207^ For TNBC BM patients, the median OS after SRS was 19.5 months.^208^ In patients treated with SRS, the 1‐year and 2‐year OS rates were 43 and 20%, respectively, with 76% of lesions showing regression.^209^ Low‐dose SRS (≤14 Gy) also contributes to effective local control.^210^ Recent meta‐analysis has shown that neoadjuvant SRS improves local control rates and reduces complication incidence.^211^ However, only a few studies have compared neoadjuvant and adjuvant SRS regimens directly, with results indicating that while the OS rate remains low, it is significantly improved.^211^ Combining RT with systemic therapies, such as SRS with tucatinib, capecitabine, and trastuzumab, is both safe and feasible for treating HER2+ BCBM.^212^

While anti‐HER2 therapy combined with RT enhances treatment efficacy, it also inevitably increases the risk of radiation necrosis. For HER2+ BCBM patients, concurrent treatment with pertuzumab and SRS has been associated with an increased risk of invasive lobular carcinoma. Nevertheless, it significantly improves both OS and local control rates.^213^ Further studies suggest that administering RT before anti‐HER2 targeted therapy may result in better intracranial PFS, although the sequence of these treatments does not impact OS.^214^ It is important to note that patients receiving SRS combined with HER2‐targeted drugs are also at higher risk for radiation necrosis.^215^, ^216^ Pyrotinib has been shown to enhance radiosensitivity in HER2+ BCBM patients.^217^ Recent clinical trial results indicate that combining pyrotinib with RT can extend mPFS (14.37 vs. 7.83 months, *p* = 0.375) and median OS (not reached vs. 36.40 months, *p* = 0.034).^218^, ^219^, ^220^, ^221^

For advanced TNBC patients, the primary treatment remains single‐agent chemotherapy or combination chemotherapy. In contrast, CDK4/6 inhibitors are the first‐line treatment for HR+/HER2− metastatic BC patients. Retrospective studies suggest that early usage of CDK4/6 inhibitors (before BM occurs) may diminish their effectiveness once BMs develop.^222^ Among 371 patients treated with CDK4/6 inhibitors, the 6‐month PFS and local control rates were 76.5 and 80.2%, respectively, while the 12‐month PFS and local control rates were 49.7 and 68.8%, respectively.^223^ Combining RT with CDK4/6 inhibitors has been demonstrated as a feasible strategy for treating BCBM.^223^ Preclinical models have shown that this combination increases CD8+ effector T‐cell infiltration in BMs, while decreasing the proportion of regulatory T‐cells (Tregs) and levels of immunosuppressive cytokines.^224^

Compared with LCBM, the treatment progress for BCBM is relatively slow. While anti‐HER2 treatment is effective for BM from HER2+ BC, including monoclonal antibodies, TKIs, and ADC, combination therapy appears to offer a better therapeutic effect. However, the increased risk of radiation‐induced brain necrosis must be considered. Immunotherapy remains relatively rare. For TNBC patients, chemotherapy remains an important treatment option.

### 3.3 Differences in efficacy and mechanisms of primary tumor and BM

As a physical and biological barrier, the BBB creates a unique microenvironment for the brain. The efficacy of chemotherapy for BM is relatively poor compared with that for primary tumors, which can be partly attributed to the low intracranial drug concentration caused by the BBB. While therapeutic antibodies were traditionally believed to be unable to penetrate the BBB, but real‐world evidence shows that anti‐PD‐L1 and PD‐1 antibodies have therapeutic effects in LCBM.^225^ Although there is a moderate consistency in HLA class 1 expression between LC and BM, nearly a quarter of patients exhibit inconsistent HLA expression. Antigen presentation loss may represent one of the many potential mechanisms for inconsistent responses to ICIs therapy.^226^ Furthermore, the heterogeneous microenvironment of tumors greatly reduces the therapeutic efficacy of ICIs.^227^ Multiple immunofluorescence and spatial transcriptomics studies revealed that ICB reduces a unique population of CD206+ macrophages in the perivascular space, which may regulate T cell entry into BM. Biomimetic codelivery strategies can potentially reverse osimertinib resistance by inhibiting macrophage‐mediated innate immunity.^228^

LC cells travel long distances and grow within the brain. Tumor cells in BM exhibit distinct characteristics, including specific molecular expressions that influence therapeutic outcomes. For example, cells with high expression of S100A9 may evade osimertinib‐induced killing and promote tumor recurrence. Mechanistically, S100A9 upregulates the expression of ALDH1A1 and activates the retinoic acid (RA) signaling pathway.^229^ Similarly, the S100A9/RAGE interaction also mediates radiation resistance in LCBM.^230^

Metabolic abnormalities are a crucial mechanism underlying chemotherapy resistance in BM. Tumor metabolism, a hallmark of cancer, plays a pivotal role in mediating various therapeutic resistances.^231^ For instance, GPX4 activates the WNT/NR2F2 signaling pathway by regulating GSTM1, leading to high glutathione consumption and consequent resistance of BM to platinum‐based chemotherapy.^232^ Warburg originally observed that cancer tissue sections in vitro utilize large amounts of glucose to produce lactate even in the presence of oxygen, a phenomenon known as aerobic glycolysis or the Warburg effect.^231^ Aldo‐keto reductase family 1 B10 (AKR1B10) in BM promotes Warburg metabolism by regulating lactate dehydrogenase, ultimately contributing to pemetrexed resistance in BM.

The brain's unique metabolic feature is the coupling of neurons and astrocytes through glutamate, glutamine, and lactate.^233^ Metabolic pathways, including glycolysis, alanine, aspartate, and glutamate metabolism, as well as arginine biosynthesis, are significantly altered in BM patients.^234^ Additionally, there are notable differences in BMs depending on the primary lesion or the timing of metastasis, such as synchronous, latent, and metachronous metastases.^235^ More importantly, metabolic disorders related to fat synthesis and decomposition are prevalent in BCBM. Fat synthesis enables tumor cells to adapt to the brain's low‐lipid microenvironment, facilitating their colonization and growth.^236^ Targeting fat metabolism in BC cell has emerged as an effective treatment strategy.^236^

The treatment of BM differs significantly from that of primary tumors. One of the most significant differences is the BBB. The BBB is dynamic during tumorigenesis, and its conversion to the blood–tumor barrier (BTB) is common. As a biophysical barrier, the BBB maintains the relative stability of the intracranial microenvironment but also limits drug penetration. Utilizing new drug carriers, nanotechnology, and physical methods to overcome the BBB may be effective treatment strategies. Additionally, the differences between tumor cells in primary tumors and BM are substantial. Variations in gene expression and metabolism might account for the differences in treatment efficacy between primary lesions and BM. Employing multiomics approaches and preclinical studies to explore the mechanisms of BM will aid in developing potential treatments.

## 4 MODELS’ ESTABLISHMENT FOR METASTATIC BRAIN TUMOR

**FIGURE 2:**
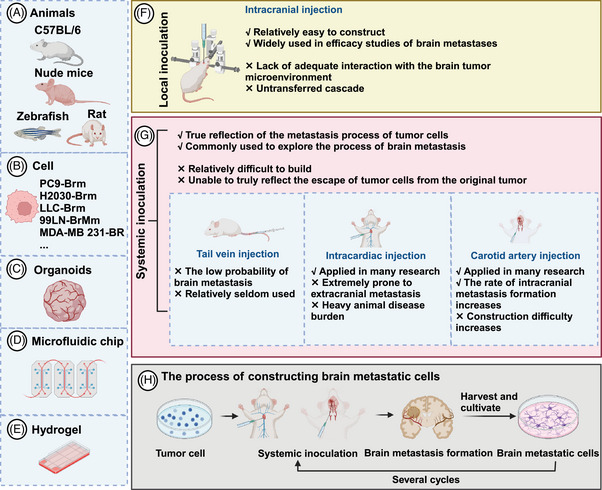
Common preclinical models in brain metastasis research. (A–E) Common models include animals (such as mice (most common), rats, zebrafish), brain‐tropism cells (often labeled with the end of Brm or BR), organoids, and microfluidic chips. (F and G) The advantages and disadvantages of local and systemic inoculation are considered to construct animal models of brain metastasis. (H) Describes the process of constructing brain‐tropism cells (also called brain metastatic cells).

In clinical trials, the inclusion of BM patients marks a significant milestone.^237^ Although BM are often analyzed as a subgroup of primary tumors, many reliable results have been obtained.^238^ However, BM, which originate from primary tumors, exhibit distinct characteristics. that necessitate their study as a distinct entity. The development of traditional cell and animal models, as well as newer models like spheroids, organoids, and tumor‐on‐a‐chip, is integral to preclinical research (Figure 2). Organoids are believed to better reflect individualized tumor characteristics and are anticipated to have a promising future.^239^, ^240^ Despite this, determining the optimal model is challenging due to their varying advantages and disadvantages. Therefore, selecting the most appropriate model based on specific research needs and cost considerations is prudent. Li et al.^241^ suggest that combining microspheres/organoids with microfluidic technology can enhance the simulation of in vivo tumor environments.

### 4.1 Cell and animal models

Cell and animal models form the foundational framework in BM research.^242^ Currently, in vitro studies often utilize commercially available tumor cells, while animal models enhance the credibility of findings in BM research.^243^, ^244^ Recently, the development of BM cell lines has gained prominence. Valiente et al.^245^ coordinated 19 laboratories to establish a panel of cells with brain tropism, providing comprehensive insights into experimental models of BM. This collaborative effort has significantly advanced BM as a distinct research field.^245^ Animal models predominantly involve mice, with additional utilization of rat and zebrafish models.^245^, ^246^, ^247^ Common models involve injecting human cells into immunodeficient mice, utilizing species‐specific mouse‐derived cells like LLC and 4T1, which facilitates robust platforms for studying BM immunotherapy.^245^ Orthotopic tumor models in animals typically encompass BC, LC, and melanoma.^245^ Various inoculation methods include systemic and local approaches.^248^ Local inoculation, which directly injects tumor cells into the brain parenchyma via a syringe, is straightforward for BM modeling but lacks adequate interaction with the brain microenvironment and fails to replicate the metastatic cascade, diminishing its relevance in BM research.^249^ Systemic inoculation, involving the introduction of tumor cells via routes such as the tail vein, carotid artery, or intracardiac injection, accurately mimics tumor cells circulation, BBB breach, and colonization in brain parenchyma. Nonetheless, systemic inoculation presents challenges like complexity, incomplete representation of tumor cells escapes from primary sites, potential extracranial tumor formation, and increased animal disease burden.^248^, ^250^

Spontaneous BM models require tumor cells to independently complete all metastatic cascade steps from spontaneously arising or orthotopically implanted tumors, faithfully reflecting BM progression.^251^ However, high costs and lengthy timelines restrict their widespread use in BM research. Patient‐derived tumor xenograft (PDX) models involve implanting tumor tissue or primary cells from patients into immunodeficient mice to retain parental tumor histopathology, molecular traits, and drug responses.^252^ Despite these advantages, PDX models face challenges such as low success rates, extended experimental cycles, and species differences, limiting their applicability in BM research.^253^

Most in vitro studies in BM employ tumor cell lines derived from primary tumors, which somewhat undermines the robustness of conclusions in BM research. Establishing a cell model of BM often relies on refined animal models. The current research paradigm involves continuously adapting tumor cells using animal models to enhance their propensity for BM. Enhancing animal model construction techniques and employing humanized mice could potentially advance preclinical BM models.^254^ Systemic therapies like chemotherapy, targeted therapy, and immunotherapy primarily rely on models of primary tumor, with scant exploration into their efficacy against BM in preclinical settings. Local treatment parameters, such as specific RT parameters, play crucial roles in influencing the trajectory of BM research.^248^, ^255^ Shi et al.^248^ summarized findings in BM cells and models concerning RT, detailing advancements from model establishment to therapeutic strategies integrating RT. Their work furnishes comprehensive and meticulous data for refining RT‐based local treatments for BM.^248^ Nonetheless, variability in specific RT parameters such as dosage and dose rate in preclinical BM models warrants greater attention.^248^

### 4.2 Organoids

Organoids are tissue analogs with defined spatial structures formed by three‐dimensional culture of adult or pluripotent stem cells in vitro.^76^ They effectively preserve molecular, cellular, and histological phenotypes of original tumors, thereby maintaining patient‐specific tumor heterogeneity, which confers unique advantages in disease modeling and precision tumor therapy.^256^ However, the complexity of brain tumor biology and the unique brain microenvironment have somewhat hindered the full development of organoid models. Bridging the gap to realistically reflect nerve–tumor interactions observed in patients remains a significant challenge for current research using conventional cell and animal models.^257^ In 2018, Bian et al.^258^ pioneered the establishment of an organoid model capable of simulating brain tumors, marking a pivotal advancement. The utilization of fetal tissue brain organoids combined with Crispr‐Cas9 technology has further enabled sophisticated brain tumors modeling.^259^ Despite several years of progress, organoids remain predominantly employed in studying brain tumors such as gliomas and meningiomas.^257^, ^260^ Recent studies by Qu et al.^244^ have explored the interaction between SCLC and astrocytes using assembloids composed of SCLC aggregates and human cortical organoids. Fitzpatrick et al.^261^ successfully developed organoids derived from BC patients with leptomeningeal metastasis. Choe et al.^262^ established a three‐dimensional in vitro model that more accurately replicates BM using tumor cells and brain organoids derived from human embryonic stem cells (metastatic brain cancer brain organoids). Quaranta and Linkous^263^ expanded on their primary brain tumor models to create an authentic in vitro model of brain development for studying BM. Currently, organoid models are increasingly employed as surrogate models for BM in vitro experiments, drawing from insights gained in brain tumor research, particularly gliomas.^264^ Understanding the biological underpinnings and the TME specific to LCBM holds the key to further advancing organoid models in this context.^263^, ^265^

To simulate the interaction between BC and the brain microenvironment, Wang et al.^266^ cocultured various BC cells with brain organoids derived from human embryonic stem cells to construct an organoid model. They discovered that MDA‐MB‐231 and SUM159PT cells could form tumor colonies within human brain tissue.^266^ Organoid model have also facilitated the development of drug screening platforms, advancing the new treatment strategies for BM.^261^, ^267^

### 4.3 Microphysiological systems

Microfluidic chips represent a powerful technical tool offering advantages such as replicating the in vivo microenvironment, low sample consumption, high automation, and seamless integration.^268^ Their capability to construct metastasis cascade models is pivotal in cancer research.^269^, ^270^ For instance, Kim's team developed a three‐dimensional microfluidic platform integrating astrocytes, brain endothelial cells (BECs), and patient‐derived NSCLC cells to simulate BM.^271^ Cancer metastasis accounts for 90% of cancer‐related deaths, underscoring the importance of constructing metastasis cascade models in microfluidic chips to study vascularization, tumor cells invasion, and simulate processes like intravasation and extravasation.^261^, ^272^ CTCs play a critical role in mediating tumor metastasis. CD44+CD74+ CTCs are prevalent in BM patients and serve as effective indicators for diagnosing BM.^273^ Microfluidic technology facilitates the enrichment of CTCs, enhancing our understanding of their role in metastasis.^274^ Moreover, microfluidic chips are frequently employed to simulate the BBB.^271^, ^275^ For instance, Lim et al.^276^ developed a choroid plexus‐on‐a‐chip utilizing oscillatory flow to mimic the human brain choroid plexus, offering a novel model for studying BM.

Using microfluidic technology, Lim et al.^276^ integrated features such as capillaries, epithelial layers, and secretory components to accurately simulate the characteristics and dynamics of the human brain choroid plexus.

### 4.4 Hydrogel model

The hydrogel model serves not only to study the interaction between BC cells and the extracellular matrix (ECM) but also to reversibly simulate the dormancy of BC cells in the brain.^277^, ^278^, ^279^, ^280^, ^281^ Yakati et al.^282^ cultured BC cells on soft hyaluronic acid (HA) hydrogels (0.4 kPa), which mimicked a dormant phenotype, and on stiff HA hydrogels (4.5 kPa), which mimicked a proliferative phenotype. They found that cells on soft HA hydrogels exhibited chemotherapy resistance through the p38–SGK1 signaling pathway.^282^

Model construction of BM is complex and challenging, which hinders the progress of preclinical research to some extent. Currently, the most widely used models in preclinical studies are cell and animal models. Cell models are primarily derived from brain tropism cells. The methods for establishing animal models are diverse, with researchers often weighing the pros and cons based on specific research goals. Additionally, the organoid model, despite its difficulty and cost, is noteworthy because it closely resembles clinical reality and can significantly enhance precise, individualized treatment. Moreover, tumor dormancy is a distinctive feature of BM. The use of hydrogel models to simulate BC dormancy has also advanced preclinical research. Additionally, Li et al.^283^ developed and validated a physiologically based pharmacokinetic model to predict plasma and central nervous system pharmacokinetics using a four‐compartment permeability finite brain model.

## 5 ADVANCES IN THE APPLICATION OF MODERN TECHNOLOGY IN METASTATIC BRAIN TUMOR

### 5.1 Bulk RNA transcriptome

**FIGURE 3:**
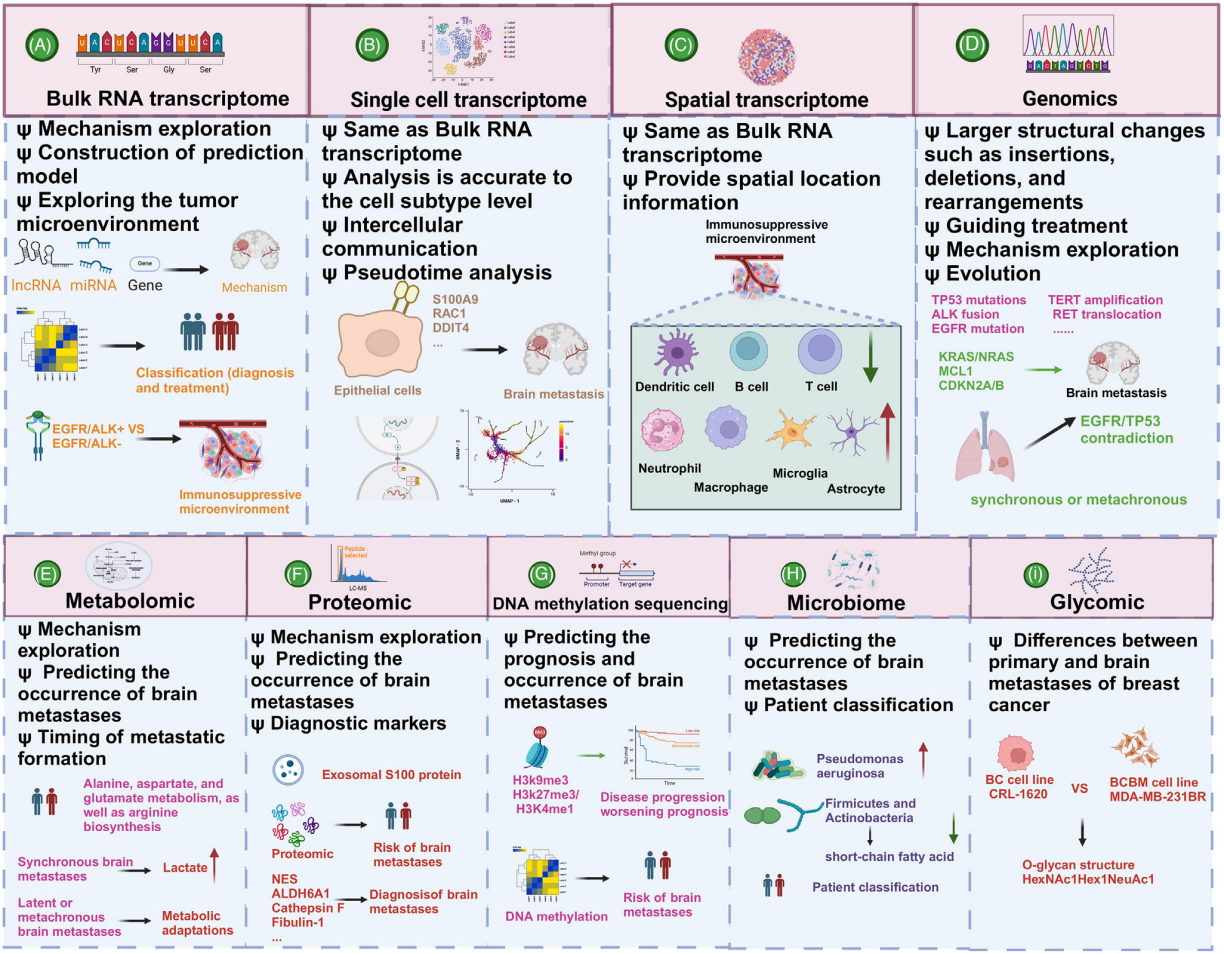
The role and the latest progress of various omics in brain metastasis. Transcriptome (bulk RNA transcriptome, single‐cell transcriptome, and spatial transcriptome), genomics is the most common omics in brain metastasis. In addition, metabolomics revealed the mechanism and timing of occurrence of breast cancer brain metastasis. Proteomics, DNA methylation sequencing, microbiome, and glycomic have also gradually developed. Although the functions of each omics are generally similar, they complement each other's strengths and weaknesses. Integrating multiple omics data are the current trend.

Bulk RNA transcriptome analysis has been a powerful tool in elucidating molecular mechanisms, past and present, owing to its cost‐effectiveness. Also, it assists in exploring the TME of BM (Figure 3A). After animal models simulate LC metastasis and obtain tissues or metastatic cells of distant metastasis (such as lymph node metastasis, bone metastasis, and BM, etc.), bulk RNA transcriptomics revealed that miR‐660‐5p may be a key driver molecule of NSCLC and distant metastasis.^284^ Similarly, the miR‐17‐5p/HOXA7 axis may induce LCBM through ferroptosis.^285^ Finding key molecules that drive BM, such as miRNAs, lncRNAs, and circRNA, is also a hot topic.^199^, ^286^, ^287^, ^288^ TCR sequencing by Zhou and Chen's^289^ team showed a unique pattern of stronger oligoclonal T cell expansion, weakened CD8+TIL infiltration in BM, and CD8+TIL was an independent positive indicator of OS.

Driver gene mutations represent a major characteristic of NSCLC. Studies have consistently demonstrated that patients harboring these mutations exhibit poorer responses to immunotherapy. Consequently, gaining a deeper understanding of the immune microenvironment in NSCLC across different driver gene subtypes has become a forefront research area. Zhou's team conducted a comprehensive analysis of the TME in EGFR/ALK‐positive/‐negative LCBM.^31^ They observed a decrease in CD8+ T cells and cytotoxic lymphocytes alongside an increase in M2 macrophages, which collectively contribute to an immunosuppressive TME.^31^ This disparity underscores why patients with positive driver genes experience limited efficacy with immunotherapy. Moreover, compared with EGFR wild‐type malignant adenomas, EGFR‐mutated LCBM exhibit upregulation of multiple immune‐related pathways.^287^

Although obtaining paired paraffin‐embedded sections of LC lesions and LCBM presents challenges, such specimens are crucial for convincingly illustrating the differences between primary tumors and metastatic lesions.^31^, ^290^, ^291^ Chen et al.^292^ delved into this by analyzing paired specimens from 43 patients, revealing significant immune heterogeneity between synchronous and metachronous LCBM. The TME is more complex in the metachronous group.^292^ Additionally, conflicting findings regarding expressions and correlations of PD‐L1 between BM and LC exist.^289^, ^292^, ^293^ While PD‐L1 expression is generally low in LCBM, its relationship with the time interval of BM in NSCLC has been reported.^290^ Tsakonas et al.^294^ conducted a study using 25 paired pathological specimens to identify differentially expressed miRNAs in LCBM, aiming to uncover potential biomarkers and therapeutic targets.

Similar to LC, one of the current research trends is to discover metastasis‐related genes and predict the TME and immune checkpoints through bulk RNA transcriptomics. Xiao et al.^295^ confirmed that oxidative phosphorylation (OXPHOS) utilization is increased in BCBM patients and that the immune microenvironment is inhibitory. Based on the bulk RNA‐seq, B7‐H3 was identified as a checkpoint for T cell immunosuppression. B7‐H3 expressed in 90% of BCBM but relatively low in BMs from colorectal and renal cancers, indicating it may be a potential target for immunotherapy.^296^ Zhang et al.^297^ used transcriptomics to explore the mechanisms of organ‐specific metastasis and demonstrated that the neuroactive ligand–receptor interaction pathway plays a significant role in BCBM.

### 5.2 Single‐cell transcriptome

Single‐cell transcriptome, in simple terms, is a technology that sequences and analyzes the genome, transcriptome, and proteome of individual cells. It addresses challenges such as low sample quantity and cell variability, which are particularly relevant in research areas like stem cells, cancer, and immunity.^298^, ^299^ In BM research, single‐cell transcriptome identifies specific LC cells driving metastasis to the brain, offering potential therapeutic targets for LCBM (Figure 3B). Wang et al.^30^ utilized 11 LUAD and 10 BM samples to identify S100A9+ BM‐associated epithelial cells through pseudotime trajectory analysis, predicting BM risk with machine learning algorithms. Chen et al.^300^ employed single‐cell RNA sequencing (scRNA‐seq) on seven patients, highlighting changes in adhesion, ECM, and VEGF pathways in RAC1‐high LC cells which maybe a promoter of BM and potential therapeutic targets. Liang et al.^301^ characterized the TME via single‐cell transcriptomics, revealing a higher presence of malignant epithelial cells in LCBM, particularly those expressing high levels of DDIT4, which are associated with increased invasiveness. Wu et al.^302^ demonstrated that BM‐associated epithelial cells expressing SPP1, SAA1, and CDKN2A originate from aneuploid LC cells, mediating BM occurrence. Ongoing research continues to uncover additional genes driving BM, such as CKAP4, SERPINA1, SDC2, and GNG11, offering promising targets for treatment.^297^ Single‐cell transcriptomics uniquely enables pseudo‐time‐series and cell‐to‐cell communication analyses, providing critical insights into how BM interacts with other cells in the TME.^266^

ScRNA‐seq can explore the developmental trajectory of tumor metastasis, uncover gene functions, and provide a comprehensive understanding of metastatic development in BCBM. Xie et al.^303^ identified ILF2 as specifically related to BCBM and a possible therapeutic target through scRNA‐seq. Zou et al.^304^ conducted scRNA‐seq on mouse lesions of BCBM, identifying microglia markers and demonstrating the conservation of their proinflammatory response. Their combination of scRNA‐seq and multiple immunofluorescences confirmed the presence of immunosuppressive cells, such as FOXP3+ Tregs and LGALS1+ microglia, in the BCBM microenvironment.^304^ Additionally, integrating single‐cell and bulk RNA transcriptomics, previously unrecognized immune regulatory subtypes enriched in BMs were identified, representing potential targets for new therapeutic strategies.^304^ They also found that the PD‐1 receptor and PD‐L1/2 ligand may not be the primary immune checkpoint signaling pathways in liver and brain metastases of BC.^304^ Interestingly, Bejarano et al.^305^ focused on endothelial cells and parietal cells in the vascular system.

### 5.3 Spatial transcriptome

Spatial transcriptome analyzes gene expression data with spatial resolution, providing simultaneous insights into the spatial organization and transcriptional profiles of cells. Named the “technology of the year” by Nature Methods in 2020, this advanced technique has become indispensable in fields such as tumor biology, immune infiltration, pathology, and disease mechanisms.^306^ In LCBM, spatial transcriptomics has enabled a more detailed characterization of the TME.^307^ Zhang et al.^32^ utilized spatial transcriptomics to identify regions of immunosuppression and fibrosis (Figure 3C). Specifically, they observed decreased presence of antigen‐presenting cells, B cells, and T cells, along with increased numbers of neutrophils, M2 macrophages, immature microglia, and reactive astrocytes.^32^ These findings align with similar conclusions drawn by Liang et al.^301^ in their single‐cell transcriptome study, which highlighted increased myofibroblast cancer‐associated fibroblasts (CAFs) and enhanced angiogenic capabilities of endothelial cells.^32^, ^289^, ^301^

### 5.4 Genomics

Cancer genomics contribute to explore the molecular mechanisms underlying carcinogenesis, tumor heterogeneity, classification, and personalized treatment strategies.^308^ Despite its critical role in understanding metastatic progression, which is the primary cause of cancer‐related deaths, the genomic mechanisms driving metastasis remain poorly understood (Figure 3D).^309^ Nguyen et al.^309^ conducted a prospective genomic analysis of 25,000 patients, revealing distinct genomic characteristics in BM originating from different primary tumors such as LC, prostate cancer, and BC. LUAD BM were found to exhibit a higher frequency of TP53 mutations, TERT amplification, and EGFR mutations, but a lower frequency of RBM10 mutations.^309^ In LCBM, Huang and Smyth confirmed frequent genomic alterations in EGFR, KRAS, TP53, RB1, MYC, CDKN2A, CDKN2B, STK11, NKX2‐1, KEAP1, MAPK, PI3K, mTOR, and cell cycle‐related pathways.^310^, ^311^ A systematic review and meta‐analysis involving 9058 NSCLC patients identified ALK positivity and RET translocation as the most prevalent genomic alterations, accounting for 34.9 and 32.2%, respectively.^4^ The genomic profile may indicate a predisposition for tumor metastasis. For instance, KRAS/NRAS mutations are particularly associated with brain and bone metastases, whereas PD‐L1 expression and TP53 mutations may influence metastasis to other organs in NSCLC.^287^, ^312^ Analysis of 3600 cases of SCLC BM revealed frequent alterations in PTEN.^313^

Chromosomal instability is closely associated with the metastatic burden of LUAD and BC.^309^ Genome sequencing has revealed that most mutations are acquired before metastasis.^309^ For instance, new copy number events in the MCL1 gene were observed in 75% of cases, may promote BM.^314^ Deletions of CDKN2A/B and alterations in the cell cycle pathway were also enriched in BM.^315^ Recent evidence suggests that LCBM exhibit increased genomic instability and complexity, potentially due to deficiencies in homologous recombination or other mechanisms involving somatic copy number changes during metastasis.^316^ However, whole exome sequencing has shown that the overall mutational signature remains relatively consistent between primary tumors and BM. Key driver genes such as EGFR and TP53 appear to be largely conserved across these tumor stages.^317^ Furthermore, genome sequencing has delineated distinct mutational signatures in synchronous versus metachronous LCBM, underscoring the potential benefits of CSF biopsy where alterations in EGFR and TP53 are frequently observed.^318^ Deng et al.^319^ also highlighted the significance of CSF genomics.

During the progression of BC, primary tumors and BMs often exhibit discordant genetic mutations.^105^, ^320^, ^321^, ^322^ Comparisons between BMs and primary tumors have revealed genes with increased mutation frequency in BMs, including TP53, ATR, and APC (in LUAD); ARID1A and FGF10 (in SCLC); PIK3CG, NOTCH3, and TET2 (in lung squamous cell carcinomas); ERBB2, BRCA2, and AXL1 (in BC).^323^

Bhogal et al.^324^ analyzed 822 BCBM tissues and identified nine common structural rearrangement gene mutations, with CDK12 being the most prevalent. Lu et al.’s^325^ genomic mutation analysis of the Chinese population revealed that somatic mutations in TP53 (82%), PIK3CA (35%), and MLL2 (22%) were the most common. The most frequent copy number alterations were HER2 (64%), RAD21 (36%), and CCND1 (32%).^325^ Genome detection by ctDNA showed that ESR1 and BRCA2 were more commonly mutated in BCBM patients.^69^ Huang et al.^326^ conducted a meta‐analysis of genomic information from 37,218 BCBM patients and confirmed that the mutation incidence of ESR1, ERBB2, EGFR, PTEN, BRCA2, and NOTCH1 in BM patients was significantly higher compared with those with extra‐brain metastases.^327^

Mutated genes in BMs are primarily involved in regulating gene transcription, cell cycle, and DNA repair processes.^321^ Genome sequencing of BMs from various primary tumors revealed mutations in genes associated with the cyclin‐dependent kinase (CDK) pathway, including CDKN2A, RB1, CCND2, and CCND3, as well as genes related to the PI3K–AKT–mTOR and MAPK pathways, such as BRAF, KRAS, and MAP2K1.^328^, ^329^ Additionally, potential targets for BCBM, such as PARP and ATM, also offer new therapeutic prospects.^321^, ^329^, ^330^

### 5.5 Metabolomics

Metabolically flexible disseminated tumor cells utilize nutrients from distant organs to sustain their survival and growth (Figure 3E).^331^ This adaptability underscores the complex metabolic alterations that occur during metastasis. In a study examining metabolome changes in 88 BCBM patients, significant alterations were observed in metabolic pathways, including alanine, aspartate, and glutamate metabolism, as well as arginine biosynthesis. A predictive model for BM occurrence constructed using 15 metabolites achieved a remarkable accuracy of 96.6%.^234^ Bulk RNA‐seq further revealed that genes related to metabolic stress pathways, particularly those involved in glycolysis and OXPHOS, were upregulated in BCBM and LCBM compared with their primary cancers.^295^, ^332^ This suggests that these metabolic pathways play a crucial role in the progression and sustenance of metastatic cells.

Metabolomics offers insights into the timing of metastatic formation. HER2+ BC patients can present with synchronous, latent, or metachronous BMs. Parida et al.^235^ explored the metabolic differences among these patterns. In synchronous BMs, lactate secreted by invasive metastatic cells was found to hinder innate immune surveillance and promote metastasis.^235^ Conversely, latent or metachronous BMs exhibited metabolic adaptations such as oxidizing glutamine, and maintaining cellular redox homeostasis via the anionic amino acid transporter xCT.^235^ Further experiments indicated that fragmented mitochondrial puncta in latent BM cells oxidize fatty acids to support cellular bioenergetics and redox homeostasis, facilitating metabolic reprogramming for survival.^331^ Additionally, RA receptor responder 2, a multifunctional adipokine and chemokine, is downregulated in TNBC cells and regulates rapamycin levels and triglyceride levels through the PTEN–mTOR–SREBP1 signaling pathway.^333^

Future research trends emphasize multiomics approaches to unravel metastasis cascades and develop therapeutic targets.^334^ Integrating whole exome/genome sequencing with RNA sequencing to investigate the immune microenvironment and immune checkpoint composition of tumors is essential for advancing treatment strategies.^335^

### 5.6 Proteomics

Proteomics has proven to be a valuable tool in identifying biomarkers for predicting the development, prognosis and therapeutic efficacy of BM (Figure 3F). Li et al.^336^ utilized plasma exosome proteome sequencing to analyze the protein composition of exosomes in LCBM and revealed significant heterogeneity between SCLC BM and NSCLC BM. They discovered that exosomal proteins in LCBM are predominantly calcium‐dependent/S100 proteins.^336^ Deng et al.^337^ developed a proteomic‐based predictive model for BM occurrence in patients with EGFR mutations, achieving an impressive AUC of 0.9401. Integrating proteomics with other datasets enhances our understanding of the BM microenvironment. For instance, [64Cu] [Cu (ATSM)] PET imaging combined with proteomic analysis revealed changes in protein expression related to hypoxia and oxidative stress in BM.^338^ High levels of proteins such as NES, ALdh6a1, Cathepsin F, and Fibulin‐1 were identified as emerging diagnostic markers.^339^, ^340^

Quantitative proteomic analysis based on high‐resolution mass spectrometry has revealed that tenascin C levels are significantly elevated in young mice with BM. This elevation promotes tumor cells proliferation and migration, which may explain increased propensity for BM observed in younger BC patients.^341^, ^342^

### 5.7 DNA methylation sequence

Epigenetics is believed to be closely linked to the onset, progression, and treatment response of BM.^343^ DNA methylation, a form of chemical modification that alters gene expression without changing the DNA sequence, plays a significant role in these processes (Figure 3G). Distinct methylation patterns are observed in primary LC tissues with and without BM. A comprehensive analysis of methylation profiles across normal lung tissue, primary LC, and LCBM has revealed that methylation patterns, such as H3k9me3 and bivalent marks like H3k27me3 and H3K4me1 may drive disease progression and worsen prognosis in BM.^344^ Furthermore, a model utilizing DNA methylation data achieved an impressive AUC value of 0.94 for predicting the risk of BM.^345^

Methylation array analysis revealed that hypermethylation of RP11‐713P17.4, MIR124‐2, and NUS1P3, as well as hypomethylation of MIR3193, CTD‐2023M8.1, and MTND6P4, may be linked to the BM metastasis of BC.^346^ Additionally, dysregulation of DNA methylation has been observed in both primary tumors and BMs.^346^, ^347^ Moreover, experiments have shown that the positive rate of the m6A reader IGF2BP3 increases with BC progression and is significantly associated with BCBM.^348^

### 5.8 Microbiomes

Microbiomes have emerged as novel tumor markers and a focal point in cancer research over the past decade. The microbiome, comprising intestinal microorganisms, other mucosal organ microorganisms, and intratumoral microorganisms, influences tumor progression through modulation of tumor growth, inflammatory responses, immune evasion, genomic stability, and resistance to treatment.^349^ The concept of the gut–brain axis has spurred investigation into the microbiome's role in BM (Figure 3H).^350^, ^351^ Dong et al.^352^ investigated alterations in intestinal and sputum microbiota in NSCLC with distant metastasis, revealing distinct microbial compositions across different metastatic sites, notably elevated Pseudomonas aeruginosa levels in BM patients. Jiang et al.^353^ analyzed the intestinal microbiome and fecal short‐chain fatty acid levels in healthy individuals, early LC patients, and those with LCBM. They identified reduced Firmicutes and Actinobacteria associated with altered short‐chain fatty acid content, suggesting an impact on lipid metabolism and possibly influencing LCBM occurrence.^353^ A model based on the microbiome for distinguishing BM patients achieved an impressive AUC of 0.88.^353^ Nonetheless, the microbiome remains a cutting‐edge and underexplored area in BM research. Most findings are presented in microbiome maps, and the underlying mechanisms yet to be fully elucidated. Robust evidence is still needed to clarify the microbiome's exact role in BM.

### 5.9 Glycomics

Glycomics is a discipline focused on studying the structure and function of sugar chains. As a new field emerging after genomics and proteomics, glycomics offers fresh insights into research areas such as diseases, cancer, and immunity.^354^ Alterations in glycosylation play a crucial role in BC development (Figure 3I). Onigbinde et al.^355^ reported that the isomers of the O‐glycan structure HexNAc1Hex1NeuAc1 exhibited significant changes across BC cell lines CRL‐1620 and the BCBM cell line MDA‐MB‐231BR. These findings highlight the potential of glycomics in understanding BC progression and BM.

The integration of various omics, leveraging their complementary advantages, represents a significant avenue to unravel the invasion patterns and immune landscapes for BM.^356^, ^357^, ^358^ By elucidating the key molecules and cell subpopulations driving BM, monitoring the dynamics of metastatic tumor cells, and exploring interactions between tumor cells and components within metastatic lesions, we can gain insights into the mechanisms of BM and advance clinical applications.^266^ Comparative analyses between primary tumors and BM, different metastatic sites, intracranial primary tumors, and synchronous/metachronous BM provide multifaceted perspectives essential for unraveling the complexities of BM. Currently, joint analyses of bulk RNA transcriptomes and publicly available single‐cell transcriptomes are mainstream, facilitating comprehensive investigations. TP53 mutations were notably more prevalent in BM from LUAD.^309^ Alvarez‐Prado et al.^335^ employed whole exome/genome sequencing and RNA sequencing of immune cell populations to delineate the immune genomic landscape in LCBM and BCBM. This study highlighted the impact of TP53 mutations on immune profiles, including increased tumor mutational burden and neoantigens, enhanced tumor proliferation, and altered immune cell infiltration patterns.^335^ Moreover, despite a modest overlap in differentially expressed genes between BM cell models and patient tissues, validating the relevance of these models remains essential for faithfully representing BM patients’ realities in preclinical settings.^359^ Exploring solutions such as organoid models may enhance fidelity and relevance in preclinical research. Ultimately, enhancing interdisciplinary communication, fostering collaborations spanning preclinical to clinical data integration, and advancing understanding of the biological underpinnings of BM are critical pursuits. These efforts aim to leverage patient‐derived transcriptomic, proteomic, and metabolomic profiles to elucidate diverse prognostic subtypes and inform personalized therapeutic strategies.

## 6 THE MECHANISM OF BM FROM LC AND BC

### 6.1 Break away from the original tumor and break through the ECM

**FIGURE 4:**
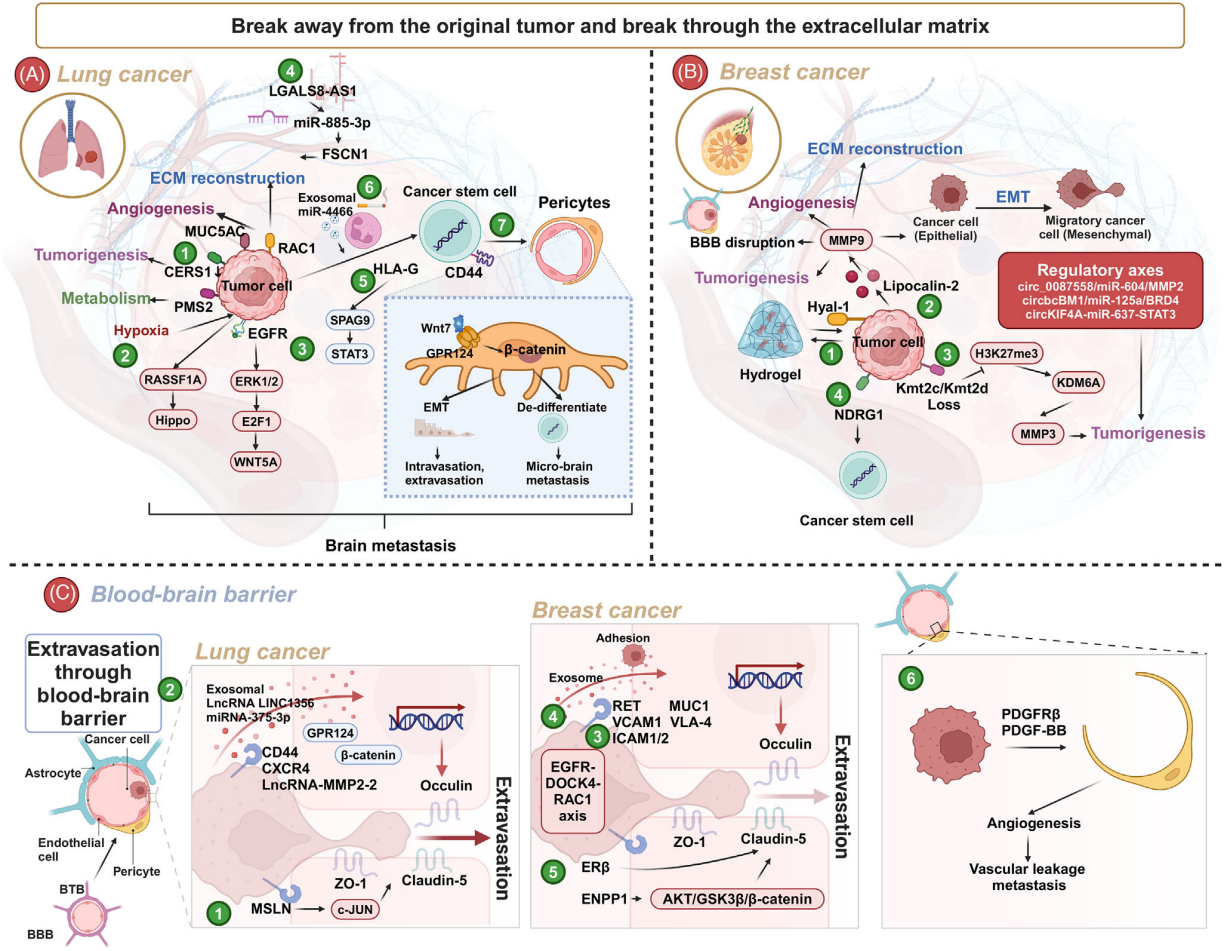
Mechanism of metastatic brain metastasis. (A‐B) Break away from the original tumor and break through the extracellular matrix. (A) Lung cancer. (A①) Genes such as RAC1, PMS2, MUC5AC regulate ECM, metabolism, tumorigenesis, and angiogenesis to promote brain metastasis. (A②) Hypoxia stimulates the RASSF1A/kinase Hippo pathway, exacerbating the formation of brain metastasis. (A③) EGFR mutation promotes brain metastasis through ERK1/2–E2F1–WNT5A. (A④) LGALS8–AS1 targets miR‐885‐3p to mediate the expression of FSCN1, ultimately leading to the formation of brain metastasis. (A⑤) HLA‐G expression is increased, and it acts on adjacent tumor cells through SPAG9/STAT3 to promote their self‐renewal ability and growth. (A⑥) CD44+ lung cancer stem cells derive vascular pericytes, form metastatic niches, and migrate across the endothelium by mediated GPR124. (A⑦) Nicotine induces neutrophils to produce exosomal miR‐4466, promoting stemness and invasion properties of lung cancer cells. (B) Breast cancer. (B①) Hyal‐1 produces hydrogel to wrap and promote tumor cells growth. (B②) Lipocalin‐2 regulates MMP9 and exerts various functions such as angiogenesis, ECM reconstruction, BBB disruption, and tumorigenesis. (B③) The loss of Kmt2c/Kmt2d promotes tumor growth through the H3K27me3–KDM6A–MMP3 axis. (B④) High expression of NDRG1 is associated with stemness and promotes tumor growth. (C) Extravasation through blood–brain barrier. (C①‐②) Lung cancer. (C①) MSLN‐overexpressing cells achieve brain metastasis by enhancing the ability to penetrate the BBB. Mechanistically, MSLN facilitates the expression and activation of MET through the c‐Jun N‐terminal kinase (JNK) signaling pathway. (C②) Exosomal LncRNA LINC01356 and miR‐375‐3p derived from brain metastatic cells, as well as Lnc–MMP2‐2‐overexpressing or CXCR4+ tumor cells, promote the occurrence of brain metastasis by inhibiting the tight junctions of the BBB (including Claudin‐5, Occulin, and ZO‐1). (C③‐⑤) Breast cancer. (C③‐⑤) Tumor cells enhance BBB adhesion through RET, MUC1, VCAM1, VLA‐4, ICAM1/2, and secretion of exosomes. Meanwhile, the EGFR–DOCK4–RAC1 axis regulates cell morphology and facilitates their passage through the BBB. (C⑥) Tumor cells and pericytes promote vascular leakage and metastasis by mimicking angiogenesis through PDGFRβ and PDGF–BB.

Gene expression in primary tumors plays a crucial role in the development of BM.^359^ Researchers are actively identifying cell subtypes that drive LC metastasis. High expression of RAC1 are pivotal in adhesion, ECM interactions, and VEGF signaling pathways, ultimately contributing to BM.^300^ Amplification of PMS2 influences thiamine, butyrate, and glutathione metabolism, promoting the formation of LCBM.^360^ Conversely, downregulation of CERS1 in LCBM enhances tumor growth via the PI3K/AKT/mTOR pathway.^361^ Studies have shown that MUC5AC is linked with increased epithelial–mesenchymal transition (EMT), invasiveness, and metastatic potential in tumors.^362^ Tumor cells with heightened angiogenic capacity facilitate nutrient supply to both primary LC and BM (Figure 4A①).^363^ Interestingly, Levallet et al.^333^ proposed that hypoxia induces activation of the RASSF1A/kinases Hippo pathway, thereby enhancing YAP and NDR2 in human bronchial epithelial cells, which exacerbates BM formation (Figure 4A②). Patients harboring EGFR mutations frequently develop LCBM. Li et al.^364^ demonstrated that EGFR mutations promote BM through the ERK1/2–E2F1–WNT5A axis (Figure 4A③). LGALS8–AS1 targets miR‐885‐3p to regulate the expression of fascin actin‐binding protein 1 (FSCN1), ultimately contributing to BM formation (Figure 4A④).^365^

BC cells facilitate tumor metastasis by regulating EMT and ECM remodeling.^366^, ^367^, ^368^ Hyaluronidase Hyal‐1 contributes to tumor proliferation by forming HA coating around the cells (Figure 4B①).^369^ The secretory iron transporter Lipocalin‐2 (LCN2) promotes tumor growth and enhances ECM reorganization, angiogenesis, and BBB disruption through interaction with MMP9 (Figure 4B②).^360^, ^370^ The loss of Kmt2c or Kmt2d in BC cells results in reduced H3K27me3, which upregulates MMP3 via KDM6A, thereby facilitating BM formation (Figure 4B③).^371^ Additionally, the circRNA–miRNA–mRNA regulatory axes, such as circ_0087558/miR‐604/MMP2, circbcBM1/miR‐125a/BRD4, and circKIF4A–miR‐637–STAT3, may influence the development of BCBM.^372^, ^373^, ^374^

Tumor‐initiating cells represent a rare but crucial subpopulation within tumors, characterized by stem‐like properties that drive tumor initiation, metastasis, and resistance to chemotherapy.^375^ RNA sequencing have revealed common transcriptomic features among BM‐initiating cells from lung, breast, and melanoma origins. In paired specimens of primary LC and BM, elevated HLA‐G expression has been observed. HLA‐G interacts with adjacent tumor cells via SPAG9/STAT3 signaling to enhance their self‐renewal and growth capabilities (Figure 4A⑤).^291^, ^376^ Additionally, CD44+ LC stem cells differentiate into vascular pericytes, facilitating the formation of metastatic niches and transmigration across endothelial barriers under the influence of G protein‐coupled receptor 124 (GPR124) (Figure 4A⑥).^377^ Smoking has been identified as a significant risk factor for BM. Tyagi et al.^378^ demonstrated that nicotine induces neutrophils to release exosomal miR‐4466, thereby enhancing the stemness of LC cells and promoting their invasive properties in BM (Figure 4A⑦). Additionally, a high expression of NDRG1 is associated with stemness and is linked to BCBM (Figure 4B④).^379^

Although proteomics has identified ECM receptor interactions and collagen‐containing ECM as crucial in LCBM, research into how tumors penetrate this ECM remains limited.^336^, ^380^ HA is a key component of the ECM and plays a significant role in the metastasis of various tumors. Tumor cells mediated by HSP47 deposit collagen in the metastatic niche, which can inhibit the antitumor immune response.^381^ Zhao et al.^382^ found that plasma HA levels are associated with bone metastasis in LC. CAFs are generally considered to promote tumor metastasis by influencing the ECM. However, their role in BM appears contradictory, and more robust evidence is needed to clarify their impact.^32^, ^98^, ^289^, ^301^, ^383^ Nevertheless, due to challenges in extracting and studying CAFs, mechanistic investigations involving these cells remain relatively complex.

### 6.2 Transfer process

The process of LC metastasis to the brain is diverse, involving a lengthy journey in terms of both time and distance. It is widely accepted that the lungs, as highly vascularized organs, facilitate the passage of tumor cells to the brain through systemic circulation.^384^ In current research, there is a predominant focus on understanding the mechanisms underlying primary and metastatic lesions, whereas tracking changes specifically in BM throughout the metastatic process remains challenging. In recent years, the advent of liquid biopsies, particularly the detection of CTCs, has not only proposed a diagnostic role for CSF CTCs in BM but also enabled the tracking of the metastatic process and prediction of treatment efficacy.^385^, ^386^ Technologies like single‐cell sequencing have furthered our ability to identify stage‐specific markers in the malignant progression and metastasis of LC, which could potentially aid in intervening at various stages of metastasis to prevent or manage BM.^387^

### 6.3 Interaction with the BBB and colonization growth in brain

#### 6.3.1 LCBM

Extravasation of tumor cells across the BBB represents a critical step in the formation of BM.^388^ In the vasculature of glioblastoma and BM, genes associated with cell proliferation, angiogenesis, and ECM deposition are dysregulated, leading to significant disruptions in the integrity of physical and biochemical barriers.^389^, ^390^ CD44+ LC stem cells differentiate into perivascular cells, form metastatic niches, and traverse the endothelium mediated by GPR124. Mesothelin (MSLN), a tumor‐associated antigen expressed in various solid tumors, has been linked to the progression of multiple cancers. Xia et al.^272^ demonstrated that MSLN enhance tumor cells to penetrate the BBB and promote BM through activation of the MET pathway via the c‐Jun N‐terminal kinase signaling pathway (Figure 4C①). Exosomal LINC01356, miR‐375‐3p, lnc‐MMP2‐2, or CXCR4+ tumor cells derived from LCBM suppress tight junction proteins of the BBB (claudin‐5, occludin, and ZO‐1), thereby promoting BM (Figure 4C②).^391^, ^392^, ^393^, ^394^

**FIGURE 5:**
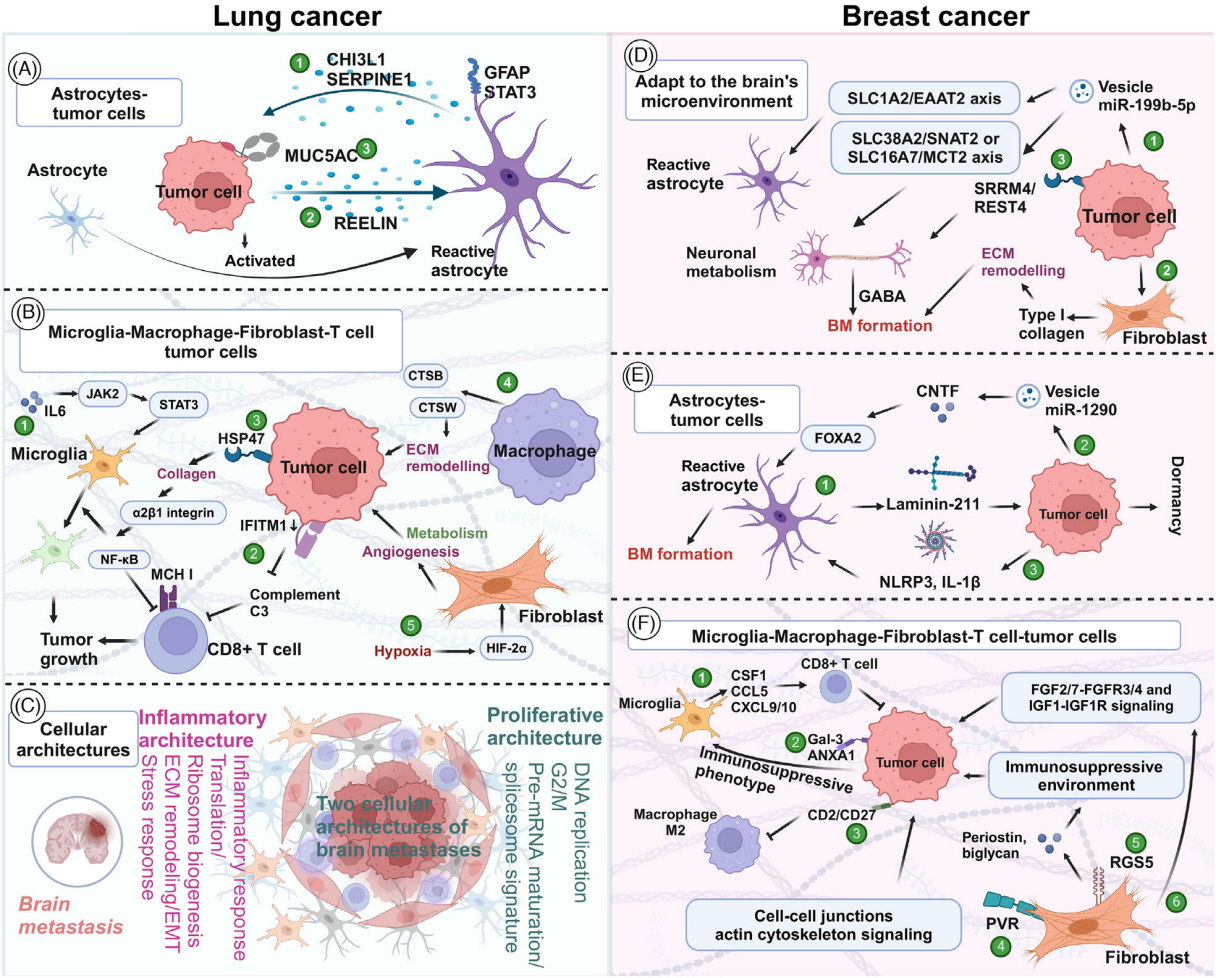
The colonization mechanism of tumor cells in the intracranial microenvironment. (A–C) Lung cancer. (A) Astrocytes–tumor cells interactions. (A①) The aggressive proliferation of metastatic brain tumor is linked to the release of CHI3L1 from p‐STAT3+ astrocytes. (A②) In SCLC, reelin is secreted to stimulate the recruitment of astrocytes, which subsequently leads to the secretion of SERPINE1 and other proteins by astrocytes, thereby promoting the growth of SCLC. (A③) Tumor cells interact with astrocytes, inducing an upregulation of MUC5AC expression in tumor cells and enhancing brain colonization. (B) Interactions between tumor cells and microglia, macrophage, fibroblast, and T cells. (B①) Increased IL6 regulates the JAK2/STAT3 signaling pathway to induce the anti‐inflammatory effect of microglia, thereby promoting the colonization of tumor cells. (B②) Low expression or loss of IFITM1 results in a decrease in complement component 3 and MHC I molecules, ultimately inhibiting the killing effect of CD8+ T cells. (B③) The HSP47–collagen axis achieves M2 polarization through the α2β1 integrin/NF‐κB pathway, leading to the upregulation of anti‐inflammatory cytokines and inhibiting the antitumor response of CD8+ T cell. (B④) High expression of cathepsins CTSB and CTSW in macrophages contributes to multiple tumor‐promoting processes including invasion and metastasis. (B⑤) Hypoxia mediates HIF‐2α, leading to CAFs undergoing a unique lineage transition. The transformed CAFs exhibit angiogenesis, trigger metabolic program rearrangement, and promote tumor cells growth. (C) Cellular architectures The proliferative BM functional archetypes are prominent in DNA replication, G2/M, and pre‐mRNA maturation as well as the spliceosome signature while the inflammatory BM functional archetypes are prominent in inflammatory response, ECM remodeling, and stress response ability. (D–F) Breast cancer. (D)Tumor cells adapt to the brain microenvironment. (D①) Tumor cells secrete vesicles rich in miR‐199b‐5p, which act on SLC1A2/EAAT2 axis to activate astrocytes and alter neuronal metabolism through SLC38A2/SNAT2 or SLC16A7/MCT2 axis. (D②) Tumor cells stimulate fibroblast secretion of type I collagen to reconstruct ECM and promote tumor growth. (D③) Tumor cells overexpress SRRM4/EST4, promoting GABA production and leading to tumorigenesis. (E) Astrocytes–tumor cells interactions. (E①) Astrocytes secrete Laminin‐211 to promote tumor cell dormancy. (E②) Tumor cells secrete vesicles rich in miR‐1290 to activate the CNTF–FOXA2 axis. (E③) Meanwhile, tumor cells increase NLRP3 and IL‐1β in astrocytes, which leads to the activation of astrocytes and promotes tumor metastasis. (F) Microglia–macrophage–fibroblast‐T cell‐tumor cells. (F①) Microglia secrete CSF1, CCL5, CXCL9/10 to activate CD8+T cells and kill tumor cells. (F②) Tumor cells overexpress Gal‐3 and ANXA1 to activate the immunosuppressive phenotype of microglia. (F③) Meanwhile, tumor cells expressing CD2/CD27 inhibit M2 type macrophage. (F④‐⑤) Fibrocytes activate cell junctions actin cytoskeleton signaling through PVR, and shape an immunosuppressive microenvironment by secreting periostin and biglycan through RGS5. (F⑥) Fibrocytes promote tumor growth through FGF2/7–FGFR3/4 and IGF1‐IGF1R signaling.

In contrast to the microenvironment of extracranial metastases, the BM microenvironment contains distinct cell types, primarily astrocytes, microglia, oligodendrocytes, and neurons.^395^ The interaction between tumor cells and the brain microenvironment facilitates breaches of the BBB, colonization, and growth. Reactive astrocytes and tumor‐associated macrophages (TAMs) are pivotal in NSCLC BM, influencing tumor progression and immune evasion. Gonzalez et al.^396^ proposed two functional prototypes of BM: proliferative and inflammatory. Proliferative BM archetypes are characterized by DNA replication, G2/M phase transition, and pre‐mRNA maturation or spliceosome signatures, whereas inflammatory BM archetypes are marked by inflammatory responses, ECM remodeling, and stress responses (Figure 5C).^396^

Astrocytes, the predominant glial cells in brain, play dual roles in different phases of BM. Initially, as integral components of the BBB, astrocytes act to impede tumor cell invasion.^397^ However, once tumor cells breach this barrier, astrocytes are exploited by tumor cells to facilitate metastatic proliferation.^398^, ^399^ Through mechanisms involving gap junctions, exosomes, and other pathways, astrocytes upregulate tumor‐related signaling, modulate the immune microenvironment, and enhance tumor metabolism, angiogenesis, and growth, thereby promoting BM.^33^, ^397^, ^400^, ^401^ Studies have confirmed that STAT3+ astrocytes foster BM in LC, BC, and melanoma by influencing both innate and adaptive immune responses.^400^ Recently, Dankner et al.^400^ demonstrated that aggressive BM proliferation correlates with CHI3L1 released from p‐STAT3+ astrocytes (Figure 5A①). In SCLC, reelin secretion stimulates astrocyte recruitment, prompting astrocytes to secrete SERPINE1 and other proteins that enhance SCLC growth in brain (Figure 5A②).^244^ Emerging research indicates that tumor cells induce astrocytes to upregulate MUC5AC, thereby promoting enhanced colonization (Figure 5A③).^362^

TAMs and microglia play crucial roles in BM. Microglia, an integral part of the brain microenvironment, are particularly significant in LCBM compared with other forms of cancer.^356^, ^402^ These microglia exhibit distinct phenotypes: M1‐type microglia promote inflammation, tissue damage, antigen presentation, and tumor cells killing, whereas M2‐type microglia support anti‐inflammatory responses, tissue repair, angiogenesis, immune suppression, and tumor progression.^402^, ^403^ Communication between tumor cells and microglia involves pathways such as DLL4–NOTCH4 and MIF–CD74, yet the full spectrum of their interactions in brain colonization remains elusive.^356^, ^404^ IL6 emerges as a pivotal mediator, with high serum levels correlating with increased BM risk by enhancing JAK2/STAT3 signaling in microglia to induce an anti‐inflammatory state conducive to tumor cells colonization (Figure 5B①).^405^ IL6 also impedes T cell function in BM and contributes to ICIs resistance.^358^ Early BM cells expressing IFITM1 stimulate microglial activation, augmenting CD8+ T cell cytolytic activity against tumor cells (Figure 5B②).^406^ Furthermore, microglia express genes encoding ECM and matrix proteins, influencing both pro‐ and antimetastatic properties.^358^ Manipulating the M1/M2 microglia ratio represents a promising strategy for BM treatment, given that factors like extracellular vesicles and radiation can induce microglial transformation toward the M2 phenotype, facilitating metastasis.^407^ The HSP47–collagen axis contributes to M2 polarization via the α2β1 integrin/NF‐κB pathway, increasing anti‐inflammatory cytokine production and inhibiting CD8+ T cell responses (Figure 5B③).^381^ Additionally, Klemm et al.^358^ underscored the involvement of cathepsins CTSB and CTSW in monocyte‐derived macrophages, promoting invasion and metastasis in LCBM (Figure 5B④). Inhibiting the anti‐inflammatory macrophage phenotype has shown promise in reducing tumor growth in mouse models of BM.^408^ In primary brain tumors like glioblastoma, macrophages exhibit a dual role as both antitumor agents and promoters of tumor progression, highlighting the complexity of their function in brain microenvironments.^409^, ^410^, ^411^ The precise roles and regulatory mechanisms of macrophages in LCBM warrant further investigation.

In typical solid tumors, CAFs are often implicated in promoting metastasis through their interactions with the ECM. However, in LCBM, their role appears paradoxical and requires further investigation to elucidate their precise contributions. Studies by Wu et al.^98^ have suggested a lack of inflammatory‐like CAFs in LCBM, contrasting with the enrichment of pericytes. This observation indicates a distinct composition of the stromal cells within the BM microenvironment compared with other tumors. Zhang et al.^32^ utilized spatial transcriptomics to confirm fibrotic niches in LCBM, while Liang et al.^301^ highlighted an increase in myofibroblast‐like CAFs and enhanced angiogenic capacity of endothelial cells in these settings.^289^ These findings underscore the heterogeneity and dynamic nature of CAF populations in BM. Hypoxia has been identified as a key mediator inducing a lineage transition in CAFs mediated by HIF‐2α, leading to enhanced angiogenesis, metabolic reprogramming, and promotion of tumor growth (Figure 5B⑤).^383^ Despite these insights, the difficulty in extracting and studying CAFs poses challenges for detailed mechanistic investigations. Single‐cell transcriptome analyses have further illuminated the role of fibroblasts in BM, revealing their high expression of type I collagen genes and significant involvement in cell–cell interactions within the TME.^383^ This highlights their potential influence on ECM dynamics and cellular interactions critical for metastatic progression. In addition to CAFs, interactions between tumor cells and brain‐specific microenvironmental components such as astrocytes and microglia play pivotal roles in BM. For instance, melanoma‐derived Aβ has been shown to activate astrocytes, promoting a prometastatic, anti‐inflammatory phenotype that supports tumor survival by inhibiting microglial phagocytosis.^412^

Overall, the complexity of the BM microenvironment underscores the need for comprehensive studies to elucidate the specific molecular mechanisms and key cell populations involved in tumor cells interactions and adaptation within the brain. Clarifying these interactions holds promise for developing targeted therapies that could potentially disrupt metastatic processes specific to the brain, improving outcomes for patients with metastatic disease.

#### 6.3.2 BCBM

Before metastatic cells can migrate through the BBB, they must first establish strong adhesion with BECs. MDA‐BR cells, derived from MDA‐MB‐231 BC cells with a brain‐specific metastatic profile, exhibit larger adhesion areas and densities, which facilitate their invasion into the brain.^413^ These tumor cells overexpress adhesion molecules such as MUC1, VCAM1, and VLA‐4, which assist them bind to vascular endothelial cells and promote extravasation.^414^ Elevated RET levels in BCBM patients enhance the expression of adhesion‐related genes in luminal BC cells.^415^ Additionally, ICAM2 on tumor cells interacting with ICAM1 in choroid plexus epithelial cells, aids adhesion in blood–CSF barrier (Figure 4C③).^416^ BC‐derived exosomes can reduce the deadhesion strength between tumor cells and BECs (Figure 4C④).^417^ Once they cross endothelial cells, tumor cells also adhere strongly to pericytes.^418^

Tumor cells employ various mechanisms to cross the BBB, including manipulating tight junction proteins and altering their own morphology. For instance, breast tumors secrete ENPP1, which disrupts BBB integrity by interfering with insulin signaling and the AKT/GSK3β/β‐catenin pathway.^419^ Estrogen receptor β promotes the expression of the tight junction protein claudin‐5, while its selective agonist, diarylpropionitrile, can inhibit BBB crossing of HER2+ BC and TNBC cells (Figure 4C⑤).^420^ Additionally, endothelial cells activate the EGFR–DOCK4–RAC1 axis, which modifies their morphology and induces TNBC cells to adopt a mesenchymal‐like phenotype, facilitating extravasation.^421^ miRNAs and other proteins also contribute to tumor cells adhesion and BBB traversal.^422^

Pericytes, along with endothelial cells, are crucial components of the BBB and play a significant role in the metastatic process. Tumor cells can recruit pericytes to promote angiogenesis and disrupt their placement within the vascular niche, leading to increased vascular leakage and facilitating metastasis.^423^ The mechanisms through which brain pericytes influence BBB invasion and the formation of metastatic niches are still not fully understood, but paracrine signaling involving PDGFRβ and PDGF‐BB may influence pericyte behavior and contribute to metastatic progression (Figure 4C⑥).^423^

While many studies emphasize that crossing the BBB is necessary for formation of BCBM, research suggests alternative pathways. BC cells can bypass the BBB by migrating through extracranial pathways, utilizing blood vessels that connect the vertebral or skull bone marrow with the meninges. This alternative route allows cells to form leptomeningeal metastases without directly crossing the BBB.^424^

Tumor cells adapt to the unique brain microenvironment to support their growth and survival. In nutrient‐deficient CSF, BC cells often interact with macrophages to enhance their survival. These tumor cells induce macrophages to produce glial‐derived neurotrophic factor, a prosurvival neurotrophin that helps them endure the challenging environment.^424^ Additionally, BC cells release extracellular vesicles containing miR‐199b‐5p, which disrupts brain metabolism by interfering with astrocyte through the SLC1A2/EAAT2 axis and affects neuronal metabolism via the SLC38A2/SNAT2 or SLC16A7/MCT2 axis (Figure 5D①). Through these mechanisms, tumor cells effectively hijack and alter brain metabolism to support growth.^233^ Furthermore, BC cells influence the ECM to better adapt to the brain microenvironment, reshaping the collagen‐rich ECM through the secretion of type I collagen by CAFs (Figure 5D②). This ECM remodeling facilitates the adaptation and proliferation of metastatic cells within the brain.^425^

BC cells also adapt to the brain microenvironment by interacting closely with neurons. Upon contact with neurons, tumor cells express genes related to neurotransmitter receptors and neuronal synaptic mediators, evolving into GABAergic‐responsive BMs. They become reliant on paracrine signaling of GABA from nearby neurons to survive and thrive in the intracranial environment.^426^ Increased expression of SRRM4/REST4 in BC cells enhance neurotransmitter and synaptic signaling pathways, allowing metastatic cells to maintain growth and function even in nutrient‐deficient conditions in the brain (Figure 5D③).^427^ This ability to utilize neurotransmitter signals for survival underscores the complex interplay between tumor cells and the neural components of the brain microenvironment, facilitating the persistence and expansion of BMs.^427^

Inflammatory activation of peritumoral astrocytes plays a crucial role in the development of BCBM, influencing tumor cells dormancy and progression.^428^ After invading the brain, tumor cells often localize near astrocytes.^429^ Laminin‐211, deposited by astrocytes, contributes to tumor cells dormancy by promoting the binding of dystroglycan receptors to yes‐associated protein (Figure 5E①).^429^ Additionally, extracellular vesicles released by BC cells are abundant in miR‐1290, which enhances the secretion of the cytokine CNTF by inhibiting the FOXA2 transcriptional repressor. This process activates astrocytes, fostering mammosphere formation and tumor progression (Figure 5E②).^430^ Moreover, BC cells induce the upregulation and activation of NLRP3 and IL‐1β in astrocytes, supporting tumor growth (Figure 5E③).^428^

Microglia exhibit a dual role in the development of BCBM. In mouse models of BM, microglia can limit the formation of BMs through their antitumor activity.^254^ Mechanistically, microglia upregulate and secrete proinflammatory cytokines, such as CSF1, CCL5, CXCL9, and CXCL10. These cytokines activate natural killer cells and T cells, promoting tumor killing (Figure 5F①).^254^ Conversely, Gal‐3 and ANXA1 facilitate BC cell migration and invasion by promoting an immunosuppressive phenotype in microglia. This phenotype impairs the proinflammatory response associated with the interferon‐related pathway, supporting tumor cells growth in the brain (Figure 5F②).^431^, ^432^ Targeting and modulating the immune phenotype of microglia with specific drugs or therapeutic strategies holds potential for effective treatment of BCBM.^433^

Preclinical models indicate that the reduced presence of myeloid cells in the aged brain, including microglia and infiltrating macrophages, is associated with a decreased formation of BMs.^341^ This finding helps explain the increased incidence of BMs observed in younger BC patients.^342^ Additionally, the overexpression of CD2/CD27 has been shown to activate the nitrogen metabolism pathway, inhibit M2 macrophage polarization, and consequently reduce BCBM (Figure 5F③).^434^ Modulating macrophage phenotype through advanced techniques such as nanotechnology represents a promising therapeutic approach.^435^

Similar to LC, the microenvironment of BCBM is characterized by immunosuppressive and fibrotic properties.^295^ CAFs are a primary component of this environment often exhibiting high expression of the poliovirus receptor (PVR).^436^, ^437^ HIF1α upregulates fucosyltransferase 11, which fucosylates PVR, enhancing cell–cell junctions and actin cytoskeleton signaling thereby promoting the invasiveness of BC cells (Figure 5F④).^436^ Additionally, RGS5+ CAFs contribute to an immunosuppressive environment by secreting periostin and biglycan (Figure 5F⑤).^304^ Single‐cell transcriptome analysis reveals that tumor fibroblasts facilitate tumor cells growth via the FGF2/7–FGFR3/4 and IGF1–IGF1R signaling pathways (Figure 5F⑥).^304^

In BCBM, CCL2 can be spontaneously generated by BC cells through NF‐κB, TNFα, and other pathways and can also be generated by tumor cells stimulated by microenvironment, or by stromal cells. Exosomal CCL2 secreted by tumor cells primarily accumulates in the primary tumor, with a small amount being taken up by CCR2+ myeloid‐derived suppressor cells and CCR2+ NK cells, promoting tumor metastasis.^438^ Additionally, small extracellular vesicles from brain organoids enhance the stemness and mesenchymal phenotype of BC cells, encouraging them to secrete MCP‐1, IL‐6, and IL‐8 to better adapt to the brain microenvironment.^439^

BMOR is a lncRNA abundant in BM cells and the brain itself. It targets IRF3 and inhibits TNF‐α signaling through NF‐κB, IFN‐α, and IFN‐γ pathways, facilitating immune escape of tumor cells.^78^ Additionally, overexpression of C‐Met in tumor cells enhances the secretion of cytokines, including CXCL1/2, G‐CSF, and GM‐CSF, which supports the self‐renewal of cancer stem cells by the secretion of neutrophil LCN2.^440^

### 6.4 Metastatic niche and immune regulation

After traversing a long journey, tumor cells arrive at distant sites and integrate into new environments, establishing a premetastatic niche.^380^, ^441^ This process involves complex interactions between tumor cells and various components of the microenvironment, ultimately facilitating their survival and growth in brain. Tumor cell‐derived exosomes play a crucial role in orchestrating the formation of this premetastatic niche. These exosomes can stimulate astrocytes to secrete cytokines such as IFN‐γ, IL‐3, IL‐5, and IL‐15. These cytokines contribute to creating an inflammatory microenvironment that supports tumor cells survival and fosters an immunosuppressive milieu that evades immune surveillance.^442^ Research by Gonzalez et al.^396^ has highlighted similarities in the metastatic niches originating from different primary tumors, suggesting that common mechanisms may underpinning the adaptation of tumor cells to the brain microenvironment, regardless of their tissue of origin. Furthermore, myeloid cells, including metastasis‐associated macrophages and dendritic cells, play pivotal roles in shaping the metastatic niche.^396^, ^443^ For instance, the loss of CSCL3 in myeloid cells leads to increased expression in CXCL10, promoting the recruitment of VISTA+ PD‐L1+ CNS‐native myeloid cells to LCBM. This recruitment creates an immunosuppressive niche that facilitates tumor immune evasion and growth.^443^ Additionally, CD44+ LC stem cells can differentiate into perivascular cells within the brain microvasculature, contributing to the formation of a metastatic niche and aiding tumor stem cells in crossing the endothelial barrier and establishing themselves within brain tissue.^377^ Overall, the formation of a premetastatic niche involves intricate interactions between tumor cells, astrocytes, myeloid cells, and other components of the brain microenvironment. Understanding these interactions at a molecular and cellular level is critical for developing targeted therapies aimed at disrupting the metastatic process and improving outcomes for LCBM patients.

The interaction between tumor cells and brain cells contributes to a microenvironment that supports BC growth by secreting factors such as ERH, RPA2, S100A9, and nerve growth factor inducible (VGF). Ahuja and Lazar^444^ investigated these secreted factors in vitro and confirmed that brain cells release factors that create an inflammatory environment. Concurrently, BC cells secrete factors like ERH, RPA2, and S100A9, which are not normally present in the brain, thereby enhancing tumor proliferation.^444^ Additionally, VGF is linked to HER2 overexpression and TNBC characteristics, which are associated with poor prognosis in BCBM patients. BC cells secrete VGF to disrupt the BBB and activate microglia.^445^ Turker et al.^446^ examined the interaction between BC cells and the ECM of brain cells using hydrogels of thiohyaluronan. Their results demonstrated that BC cells form multicellular aggregates within the ECM, which helps sustain their activity.^446^

Immune escape may be influenced by intratumor genetic heterogeneity.^316^ For instance, EGFR‐mutated NSCLC is more prone to BM, potentially due to immune escape mechanisms. Compared with EGFR/ALK‐negative LCBM, the immune microenvironment of EGFR/ALK− LCBM is more suppressive.^31^ Furthermore, Tang et al.^401^ demonstrated that EGFR‐mutated LC cells induce reactive astrocytes to secrete IL‐11, which leads to tumor cells PD‐L1 expression and CD8+ T lymphocyte apoptosis.^401^ Meanwhile, IL‐11 also acts on GP130 to assist tumor cells in immune escape.^401^ High expression of IFITM1 can promote CD8+ T cell killing of tumors by inducing microglia to secrete complement component 3, but BM cells can effectively hide IFITM1 to achieve immune escape.^406^ Additionally, brain‐derived neurotrophic factor may drive an immunosuppressive TME by converting TAMs to a protumorigenic M2 phenotype. Most studies focus on specific stages of tumor cells progression, such as detachment from the primary site, circulation, and crossing the BBB. However, Chang et al.^447^ described the role of YTHDF3 in promoting BM at various stages of BC. Understanding how tumor cells navigate to the brain and establish themselves there remains incomplete. Clarifying the key regulatory mechanisms at each stage of BM, preventing the occurrence of BM, and adopting dynamic and forward‐looking treatment strategies may reduce the occurrence of BM.

Research into the mechanisms of BM is advancing, but there are similarities in the preclinical research of both BC and LC. Primary tumors research often focuses on EMT and overcome the ECM. However, there is little studies specifically on the metastasis process itself, particularly interaction between tumor cells and the brain microenvironment. Recent research highlights interactions between tumor cells and astrocytes, microglia, and macrophages as critical areas of focus. Nevertheless, current studies have yielded inconsistent results, showing that tumor cells and the microenvironment can either promote or suppress tumor progression. Identifying cell subtypes with metastatic advantages and understanding the interactions with the microenvironment are crucial for developing potential treatments.

## 7 BRIEF SUMMARY OF THE MICROENVIRONMENT OF METASTATIC BRAIN TUMOR

### 7.1 LCBM

Recent research has overturned the long‐held belief that the brain microenvironment is immune‐exempt. Karimi et al.^307^ elucidated distinct differences in immune landscapes between various primary tumors and BM.^290^ Understanding these immunological disparities is crucial for advancing the study of LCBM as a distinct field. Souza et al.,^448^ through a meta‐analysis of transcriptomic data, identified significant transcriptional alterations in key driver genes like CD69 and GZMA, emphasizing the immune system's role in BM of LUAD. Furthermore, reaffirmation of the immunosuppressive TME in BM has been documented.^448^

**FIGURE 6:**
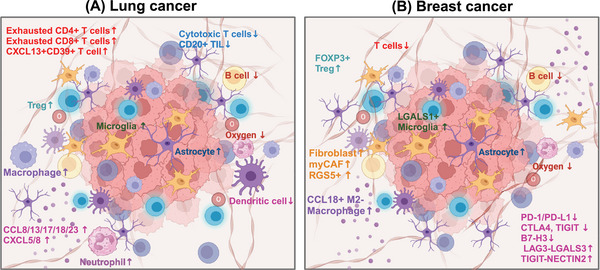
A brief summary of the tumor microenvironment of brain metastasis from lung cancer and breast cancer. Overall, brain metastases from both breast and lung cancers display a is more immunosuppressive and fibrotic microenvironment compared with their primary lesions. (A) Lung cancer. Exhausted T cells, Treg cells, macrophages, neutrophil, microglia, astrocyte, cytokines and chemokines increased. However, dendritic cells, cytotoxic T cells, and B cells decreased. (B) Breast cancer. CCL18+M2 macrophage, RGS5+ fibroblast, myCAF, FOXP3+Treg cells, LGALS1+ microglia, astrocyte increased. And dendritic cells, T cells, and B cells decreased. PD‐1/PD‐L1, CTLA4, TIGIT, B7‐H3 decreased, and the immune checkpoint that mainly mediates immune escape may be related to LAG3–LGALS3 and TIGIT–NECTIN2.

Overall, LCBM are characterized by an immunosuppressive and fibrotic niche (Figure 6A).^396^ Key features of this microenvironment include diminished immune cell subsets, reduced cytotoxic T cells, and increased populations of Treg cells and macrophages.^98^, ^291^, ^358^ Specifically, T cells in BM exhibit several changes: CD20+ TILs are decreased, CD4+ T cells display an anergic phenotype with low reactivity, and CD8+ T cells show signs of exhaustion commonly seen with chronic activation.^290^, ^358^ Wischnewski et al.,^449^ using single‐cell sequencing, identified distinct T cell subtypes associated with BM including CD39+ potential tumor‐reactive T cells expressing CXCL13. Moreover, macrophages and dendritic cells in BM also display protumor and anti‐inflammatory characteristics.^98^ The BM environment is marked by a reduction in antigen‐presenting cells, B cells, and T cells, alongside increases in neutrophils, M2 macrophages, immature microglia, and reactive astrocytes.^32^ Several cytokines and chemokines, such as TGF‐β1, Visfatin, TNF‐α, PAI‐1, IL‐2, IL‐6, IL‐7, IL‐8, and elevated levels of CCL23, CXCL5, CXCL8, CCL8, CCL13, CCL17, and CCL18, play pivotal roles in the initiation and progression of BM.^358^, ^450^ Maurya et al.^451^ summarized the chemokine cascade in brain tumors, highlighting its importance in tumor progression.

Proteomics studies have also revealed significant changes in protein expression related to hypoxia and oxidative stress in BM. Additionally, pathways related to metabolism, translation, and vesicle formation are overrepresented in metastatic tumors underlining their critical roles.^338^, ^452^ Underscoring big data, detailed descriptions of cell can be fully described, enhancing the understanding of the BM microenvironment at various stages of BM. This insight provides valuable therapeutic targets and potential strategies for intervention.^290^, ^453^

### 7.2 BCBM

Similar to LC, the microenvironment of BCBM is characterized by both immunosuppressive and fibrotic properties (Figure 6B).^295^ Xiao et al.^437^ demonstrated that BC primarily consists of immune cells, including CD4+/CD8+ T cells and M2 macrophages, whereas CAFs are predominant in BMs. Among BCBMs, only 3.8% exhibit a “hot” TME with an active immune response, whereas 73.1% display a “cold” TME, with significantly reduced TILs.^454^ In LCBM, there is an increase in AT2 cells, CD4+ T cells, and exhausted CD8+ T cells. In contrast, BCBM are marked by a high presence of epithelial cells and myCAF cells.^455^ Advanced techniques such as scRNA‐seq have identified new immune cell subsets in BMs, revealing the presence of immunosuppressive cell populations including FOXP3+ Tregs, RGS5+ CAFs, CCL18+ M2‐like macrophages, and LGALS1+ microglia.^455^

Using multiplex immunofluorescence, Griguolo et al.^456^ characterized the BM microenvironment across various BC molecular subtypes. They found distinct immune profiles for different subtypes: HR−/HER2−, HER2+, HR+/HER2−, HR−/HER2−, and HR+/HER2− BCBMs. Respectively, these subtypes exhibited variations in immune cell types, with higher levels of CD8+ lymphocytes, increased CD4+ FoxP3+/CD8+ cells, and elevated CD163+ M2‐polarized microglia/macrophages.^456^

In contrast to LC, the immune checkpoints such as PD‐1 and PD‐L1/2 are rarely expressed in CD8+ T cells and other associated cells in the BC microenvironment.^304^ Specifically, the expression of immune checkpoints including PD‐L1, CTLA4, TIGIT, and B7H3 is notably reduced in BCBMs.^454^ Additionally, immune checkpoints like LAG3–LGALS3 and TIGIT–NECTIN2 might play significant roles in facilitating immune escape.^304^

Overall, LCBM and BCBM display a more immunosuppressive and fibrotic microenvironment compared with their primary tumors. The immune cell content is reduced, and their functions are often exhausted. The immune checkpoint expression is typically decreased, and in BCBM, PD‐1/PD‐L1 may not be the primary axis of immune escape. Recent research increasingly focuses on the roles of astrocytes, microglia, macrophages, and CAFs. This summary highlights the latest research progress on BM, particularly emphasizing transcriptome data to provide up‐to‐date insights for researchers.

## 8 CLINICAL TRIALS BASED ON THE MECHANISM OF METASTATIC BRAIN TUMOR OR THE CUTTING‐EDGE TREATMENT AND MECHANISM OF TME

### 8.1 LCBM

#### 8.1.1 Inhibitors

Although the fatal weaknesses of BM have been identified through its occurrence, development, and exploration of the TME, current clinical trials typically include BM patients as a subgroup for analysis, and few trials specifically target treatments for BM. To date, inhibitors of ATR, HDAC, mTOR, PKCβ, and integrin αvβ3/αvβ5 have been combined with RT in efforts targeting BM (Table 2). In 2021, ATR inhibitors were founded to enhance the efficacy of RT in xenografts derived from LCBM.^457^ The National Cancer Institute has been investigating the efficacy of ATR inhibitors combined with RT for BM patients (NCT02589522). The PI3K–AKT–mTOR pathway has been implicated in LCBM formation.^361^ The novel PI3K inhibitor XH30 has shown promise in inhibiting orthotopic glioblastoma and BM in mice.^458^ Moreover, mTOR inhibitors have recently garnered attention (NCT00892801). While preclinical HDAC have been rarely studied in BM, they have been shown to promote tumor development and drug resistance in LC, with their inhibitors advancing to phase I/II clinical trials (NCT00838929, NCT00946673).^459^, ^460^, ^461^ Additionally, the PARP inhibitor pamiparib has demonstrated both effectiveness and safety in treating SCLC.^462^ Phase II clinical trials have indicated that veliparib combined with WBRT can inhibit the progression of LCBM.^463^ Researchers have also investigated RT combined with veliparib or apatinib for treating BM (NCT01657799, NCT03801200).

**TABLE 2:** Phase I–II clinical trials of brain metastasis in 2021–2024.

Therapy	Author	Clinical Trial ID	Year	Phase	Inclusion patients	Interventions	Patients, *N*	Results	AEs	Conclusions	References
**Lung cancer**											
**TKI alone**											
TKI	Park, Sehhoon et al.	BLOSSOM (NCT04563871)	2024	II	EGFRm NSCLC LM resistant to prior first‐ or second‐generation EGFR TKIs	Osimertinib	73	mOS: 15.6m; ORR: 51.6%; DCR: 81.3%; median LM PFS: 11.2m; duration of response: 12.6m; OS: 15.0m	NA	Significant intracranial efficacy and survival benefits of 80 mg once daily osimertinib in NSCLC patients with LMs. Daily 80 mg of osimertinib as a treatment option for EGFRm NSCLC patients with LMs, irrespective of T790M mutation status.	^464^
TKI	Lu, Shun et al.	NCT03861156	2024	II	EGFR T790M NSCLC	Befotertinib of 50 mg (cohort A) or 75–100 mg (cohort B)	140	Among patients with BM: cohort A: mOS: 18.6m; miPFS: 16.5m; 12‐month iPFS rates: 62.8%; ORR: 51.8%; cohort B: mOS: 23.0m; miPFS: 34.5m; 12‐month iPFS rates: 66.3%; ORR: 64.3%	Most common TRAEs: Cohort A: thrombocytopenia, anemia, decreased white blood cell count. Cohort B: thrombocytopenia and headache. Grade 3 or higher TRAE in the cohort A and B: 38.1 % and 50.3 %.	Befotertinib demonstrated a more profound OS benefit compared with other 3rd‐generation EGFR TKI, and the safety profile was manageable.	^465^
TKI	Morise, Masahiro et al.	VISION (NCT02864992)	2024	II	Advanced NSCLC harboring METex14 skipping (Japanese patients)	Tepotinib	4	ORR: 75%	Most common TRAEs: blood creatinine increase, peripheral edema, hypoalbuminemia, and diarrhea.	Tepotinib demonstrated robust and durable clinical activity irrespective of age or therapy line, with a manageable safety profile in Japanese patients with METex14 skipping NSCLC enrolled in VISION.	^466^
TKI	Cheng, Ying et al.	LIBRETTO‐321 (NCT04280081)	2023	II	Advanced NSCLC and BM with a centrally confirmed KIF5B/CCDC6/NCOA4‐RET fusion (post hoc case series)	Selpercatinib	8	Best overall systemic response: PR in 6/8 patients (75%); SD in 2/8 (25%); best overall intracranial response: CR in 3/8 (38%), PR in 3/8 (38%), SD in 1/8 (13%); nonprogressive disease/non‐CR in 1/8 (13%)	Grade ≥3 TRAEs: 63% cases.	Selpercatinib demonstrated clinically meaningful and durable intracranial activity in Chinese patients with BM from RET‐altered NSCLC, consistent with the global LIBRETTO‐001 trial.	^467^
TKI	Lu, Shun et al.	NCT03019276/NCT03972189	2023	I/II	ROS1 + advanced NSCLC	Unecritinib	33	I: ORR:63.9%; II: among patients with BM at baseline: ORR: 72.7%; DCR: 90.9%; miPFS: 10.1m; 6‐month PFS rate: 71.6%; 12‐month PFS rate: 30.7%	Most common grade 3 or 4 TRAEs when receiving recommended phase II dose 300 mg BID: reduced neutrophil count, elevated ALT, reduced leukocyte count.	Unecritinib is efficacious and safe for ROS1 inhibitor‐naive patients with ROS1+ advanced NSCLC, particularly patients with BM at baseline.	^468^
TKI	Jung, HA et al.	NCT04339829	2023	II	EGFRm NSCLC with BMs	Dacomitinib	30	Intracranial ORR: 96.7%, intracranial CR: 63.3%; miPFS: not reached; 12‐month and 18‐month iPFS rates:78.6% and 70.4%; 12‐month cumulative incidence of intracranial progression: 16.7%; overall ORR: 96.7%; mPFS: 17.5m	Most commonly reported grade 3 AEs: diarrhea and dermatitis acneiform.	Dacomitinib has outstanding intracranial efficacy in patients with EGFR‐mutant NSCLC with BM.	^469^
TKI	Shi, Yuankai et al.	BPI‐7711 (NCT03812809)	2022	II	Locally advanced or metastatic/recurrent EGFR T790M‐mutated NSCLC	Rezivertinib	91	ORR: 57.1%; DCR: 83.5%; mDOR: 11.1m; median intracranial DOR: 15.2m; mPFS: 10.3m; miPFS: 16.6m; mOS: 17.5m; More than or equal to one brain target lesion at baseline: intracranial ORR: 69%; intracranial DCR: 100%; median intracranial TTP: 16.5m	Most common TRAEs: decreased white blood cell count, decreased platelet count, anemia, decreased neutrophil count, increased AST, increased ALT, vomiting, and decreased appetite. Grade more than or equal to 3 TRAE: 19.9%.	Rezivertinib was found to have promising efficacy and favorable safety profile for patients with locally advanced or metastatic/recurrent NSCLC with EGFR T790M mutation.	^470^
TKI	Tan, Daniel SW et al.	NCT02108964	2022	II	EGFRm NSCLC with BM	Nazartinib	18	In patients with baseline BM: ORR: 67%; mPFS: 17m	Most frequent AEs: diarrhea, maculopapular rash, pyrexia, cough, and stomatitis.	First‐line nazartinib demonstrated promising efficacy, including clinically meaningful antitumor activity in the brain, and manageable safety in patients with EGFR‐mutant NSCLC.	^471^
TKI	Zwierenga, Fenneke et al.	POSITION20 (NL6705)	2022	II	Advanced NSCLC, harboring an EGFR ex20+ mutation (deletion and/or insertion), T790M‐	Osimertinib	25	ORR: 28%, with a mDOR of 5.3m; mPFS: 6.8m; mOS: 15.2m	Most common TRAEs: diarrhea, dry skin, and fatigue.	The POSITION20 study showed modest antitumor activity in patients with EGFRex20 + NSCLC treated with 160 mg osimertinib, with a confirmed ORR of 28% and acceptable toxicity.	^472^
TKI	Zhou, Qing et al.	NCT02590952	2022	I	EGFRm NSCLC with BM	Epitinib	72	120 mg group: ORR: 53.6%; mDOR: 7.40m; mPFS: 7.4m. 160 mg group: ORR: 40.5%; mDOR: 9.10m; mPFS: 7.40m	120 mg group: TRAEs of grade ≥3 in 120 and 160 mg group: 43.3% and 50.0%. The most frequent TRAEs in 120mg group: rash, ALT increased and AST increased. In 160mg group: rash, ALT increased and pigmentation disorder.	In patients with EGFR‐mutant NSCLC with BM, epitinib was well tolerable with a promising efficacy. According to the comprehensive assessment on safety and efficacy, 160 mg QD could be the recommended phase 2 dose.	^473^
TKI	Dagogo‐Jack, Ibiayi et al.	NCT02927340	2022	II	ALK + NSCLC	Lorlatinib	23	Intracranial ORR: 59%; miPFS: 24.6m; nine patients developed PD, including four patients with CNS progression	Most common TRAEs: hypercholesterolemia, hypertriglyceridemia, edema, cognitive effects, and mood effects.	Lorlatinib induced durable intracranial disease control in patients with CNS‐only relapse on second‐generation ALK inhibitors, suggesting that tumors with CNS‐limited progression on brain‐penetrant ALK tyrosine kinase inhibitors remain ALK dependent.	^474^
TKI	Lu, Shun et al.	NCT03909971	2022	II	Chinese patients with ALK+ advanced or metastatic NSCLC	Cohort 1: previous crizotinib/cohort 2: one ALK tyrosine kinase inhibitor other than crizotinib (±prior crizotinib)	109	In patients with brain lesions at baseline: cohort 1: intracranial ORR: 80.6%; cohort 2: intracranial ORR: 47.6%	Common TRAEs: hypercholesterolemia and hypertriglyceridemia.	Lorlatinib was found to have a robust and durable response and high intracranial ORR in previously treated Chinese patients with ALK + NSCLC.	^475^
TKI	Song, Zhengbo et al.	ChiCTR1800020262	2022	II	HER2+NSCLC with BM	Pyrotinib	78	6‐month PFS rate: 49.5%.; ORR: 19.2%; mPFS: 5.6m; mOS: 10.5m; mDOR: 9.9m	All TRAEs were grade 1–3. Most common TRAE: diarrhea.	Pyrotinib exhibited promising efficacy and acceptable safety in NSCLC patients carrying exon 20 and nonexon 20 HER2 mutations and is worth further investigation.	^476^
TKI	Cho, Byoung Chul et al.	NCT03046992	2022	I/II	EGFRm NSCLC progressed after prior EGFR‐directed TKIs	Lazertinib	40	Patients with measurable intracranial lesions: ORR: 85.7%	Most common TRAEs: rash, pruritus, and paresthesia. Serious drug‐related AEs: gastritis, pneumonia and pneumonitis.	Lazertinib 240 mg/d has a manageable safety profile with durable antitumor efficacy, including BM, in patients with advanced T790M NSCLC after previous EGFR TKI therapy.	^477^
TKI	Le, Xiuning et al.	VISION (NCT02864992)	2022	II	MET Exon 14 Skipping NSCLC (subgroup analysis)	Tepotinib	23	Systemic ORR: 47.8%; mDOR: 9.5m; mPFS: 9.5m; Intracranial disease control: achieved in 13 patients	Most common TRAEs: peripheral edema.	Tepotinib showed meaningful activity across subgroups by age, prior therapies, and BM, with a manageable safety profile and few treatment discontinuations.	^478^
TKI	Song, Zhengbo et al.	ChiCTR1800020262	2022	Prospective trial	advanced NSCLC with HER2+	Pyrotinib	27	Patients with BM: ORR: 40%	Most common TRAE: diarrhea. No grade 4 or higher TRAEs.	Pyrotinib provided antitumor efficacy with a manageable safety profile in HER2‐amplified patients with NSCLC.	^479^
TKI	Ma, Yuxiang et al.	CT‐711 (NCT03099330)	2022	I	Advanced ALK+ or ROS1+ NSCLC	TQ‐B3139	23	Intracranial ORR for brain lesions: 70.0%; miPFS: 15.9m	Most common TRAEs: grade 1–2 vomiting, diarrhea or nausea.	TQ‐B3139 was well tolerated and exhibited promising antitumor activities in patients with ALK and ROS1 + advanced NSCLC.	^480^
TKI	Wang, Pingli et al.	ChiCTR2000029062/ChiCTR2000029059	2021	Ib/II	EGFRm NSCLC with BM	Mefatinib	31	ORR: 87.1%; PFS: 12.8m; OS: 25.2m	Most common AEs: skin and gastrointestinal toxicities.	First‐line mefatinib provides durable PFS and an acceptable toxicity profile in patients with advanced EGFR‐mutant NSCLC.	^481^
TKI	Thomas E Stinchcombe et al.	ARI‐AT‐002 (NCT02706626)	2021	II	ALK+ NSCLC with BM	Brigatinib	11	mPFS: 6.3m	NA	Brigatinib has activity in ALK + NSCLC after previous next‐generation ALK TKIs.	^482^
TKI	Hiroyuki Yamaguchi et al.	OCEAN (jRCTs071180017)	2021	II	RT‐naive CNS metastasis from sensitizing EGFR + NSCLC	Osimertinib	39	BM response rate: 66.7%; median BM‐related PFS: 25.2m; 1‐year OS rate: 61.9%; 2‐year OS rate: 52.0%; median BM‐related PFS of 19‐del and 21‐L858R: 31.8m and 8.3m	Most common AE: anemia.	Osimertinib is effective in patients with CNS metastasis, especially for EGFR‐sensitizing mutation of exon 19 deletion.	^483^
TKI	Vivek Subbiah et al.	LIBRETTO‐001 (NCT03157128)	2021	I/II	RET fusion + NSCLC with BM	Selpercatinib	80	Intracranial mPFS: 13.7m	NA	Selpercatinib has robust and durable intracranial efficacy in patients with RET fusion + NSCLC.	^484^
TKI	Inger Johanne Zwicky Eide et al.	TREM (NCT02504346)	2021	II	NSCLC with BM resistant to prior EGFR‐TKIs	Osimertinib	48	ORR: 10%; T790M+ group: 6 months CNS‐progression vs. non‐CNS‐progression 0 vs. 17%. 12 months 13 vs. 40% T790M‐ group: 6 months CNS‐progression vs. non‐CNS‐progression 39 vs. 31%	NA	Other treatment options should be considered for EGFR‐TKI relapsed T790M‐negative patients with BM.	^485^
**Chemo**											
Chemo	Fan, C et al.	ChiCTR1800016615	2024	II	EGFRm NSCLC LM	Intrathecal pemetrexed	132	mOS: 12m; response rate: 80.3%	Most common AE: myelosuppression	This study further showed that IP is an effective and safe treatment method for the EGFR‐TKI‐failed NSCLC‐LM.	^486^
**ADC**											
ADC	Yu, Helena A et al.	HERTHENA‐Lung02 (NCT04619004)	2023	II	Advanced EGFRm NSCLC	Patritumab deruxtecan (HER3‐DXd)	72	CNS ORR: 33.3%; best CNS responses of SD: 43.3%; best CNS responses of PD: 13.3%; DOR:8.4m	Most common grade ≥3 TEAEs: hematologic toxicities: thrombocytopenia and neutropenia.	After tumor progression with EGFR TKI therapy and PBC in patients with EGFRm NSCLC, HER3‐DXd once every 3 weeks demonstrated clinically meaningful efficacy with durable responses, including in CNS metastases.	^165^
**Combination**											
**TKI combination**											
TKI + anti‐VEFG	Lee, Youngjoo et al.	NCT03126799	2023	II	EGFRm advanced NSCLC	Erlotinib + bevacizumab vs. erlotinib	59	Erlotinib + bevacizumab group: mPFS: 18.6m; cumulative incidence of CNS progression at 12 and 24 months: 4.4 and 6.8%; rate of CNS progression at 12 months and 24 months: 4.8 and 18.4%. Erlotinib group: mPFS: 10.3m; cumulative incidence of CNS progression at 12 and 24 months: 15.1 and 32.5%; rate of CNS progression at 12 months and 24 months: 23.1 and 63.7%	Most common AEs: skin rash, diarrhea, paronychia.	Although it was not statistically significant, a trend to improvement in PFS was observed in patients with erlotinib + bevacizumab compared with erlotinib alone.	^487^
TKI + TKI	Yang, Guangjian et al.	PATHER2 (chiCTr1900021684)	2022	II	HER2‐mutated or HER2‐amplified metastatic NSCLC	Pyrotinib + apatinib	13	ORR: 53.8%; mPFS: 6.7m	Most common TRAEs: diarrhea, hypertension, anorexia, nausea, and oral mucositis. Grade 3 TRAEs: diarrhea and hypertension. No grade 4 or 5 TRAEs.	Pyrotinib + apatinib demonstrated promising antitumor activity and a manageable safety profile in HER2‐mutated or HER2‐amplified metastatic NSCLC patients.	^488^
**Immunotherapy combination**											
ICI + anti‐VEFG + Chemo	Watanabe, Satoshi et al.	NEJ043 (jRCTs 031190066)	2024	II	EGFRm NSCLC after TKI treatment failure	Atezolizumab, bevacizumab, carboplatin, and paclitaxel (ABCP)	19	Among patients with BM at baseline: mOS: 12.9m; PFS: 5.7m	Most common AE: hypoalbuminemia. No treatment‐related death.	Although this study did not meet the primary endpoint, ABCP showed clinically meaningful efficacy in EGFRm NSCLC patients.	^489^
ICI + chemo	Nadal, Ernest et al.	Atezo‐Brain, GECP17/05 (NCT03526900)	2023	II	Advanced nonsquamous NSCLC with untreated BM	Atezolizumab + carboplatin and pemetrexed	40	Overall, 12‐week PFS rate: 62.2%; miPFS: 6.9 m; intracranial response rate: 42.7%; systemic mPFS: 8.9m; systemic response rate: 45%; mOS: 11.8m; 2‐year OS rate: 27.5%	Most common neurologic events: headache, dizziness, CNS disorder‐others. No grade 5 AE during the first 9 weeks.	Atezolizumab + carboplatin and pemetrexed demonstrates activity in patients with advanced nonsquamous NSCLC with untreated BM with an acceptable safety profile.	^137^
ICI + chemo	Hou, Xue et al.	CAP‐BRAIN (NCT04211090)	2023	II	Advanced nonsquamous NSCLC with BMs	Camrelizumab + pemetrexed and carboplatin	45	miPFS: 7.6m, median overall PFS: 7.4m, mOS: 21.0m FAS: intracranial ORR: 46.7%; extracranial ORR: 42.2% EAS: intracranial ORR:52.5%; extracranial ORR: 47.5%	Most common TRAEs of grade 3 or higher: neutrophil count decrease and anemia.	Camrelizumab + pemetrexed and carboplatin was found to have an activity with manageable toxicity and to improve cognitive function and quality of life for patients with nonsquamous NSCLC with BMs in the first‐line setting.	^340^
**ADC combination**											
ADC + chemo	Tanimura, Keiko et al.	jRCTs071180048	2023	II	Advanced NSCLC with measurable asymptomatic BM	Ramucirumab and docetaxel (DOC/RAM)	25	mPFS: 3.9m; miPFS: 4.6m; mOS: 20.9m; ORR: 20%; DCR: 68%	Most common grade 3 or higher toxicities: neutropenia. Neither intracranial hemorrhage nor grade 5 AEs.	No clinical concerns were identified with DOC/RAM for NSCLC with BM.	^490^
ADC + TKI	Hiroyasu Kaneda et al.	RELAY‐Brain (jRCTs2051190027)	2021	Ib	NSCLC patients with asymptomatic BM	Ramucirumab + erlotinib vs. ramucirumab + osimertinib	6	ORR: 83.3%	Most common TRAEs of any grade: rash, dry skin, stomatitis, diarrhea, paronychia, ALT increased, epistaxis, and edema.	Ramucirumab in combination with erlotinib or osimertinib showed safety for EGFRm NSCLC with BM.	^491^
**RT**											
**RT alone**											
RT	Li, Zhuoran et al.	NCT06289023	2024	II	LCBM	Hippocampal avoidance whole‐brain radiotherapy with a simultaneous integrated boost (HA–WBRT–SIB)	40	mOS: 14.8m; mPFS: 6.7m; miPFS: 14.8m	Common AEs: nausea, dizziness, headaches, hair loss. No grade 3 toxicities.	HA–WBRT–SIB is of efficiency and cognitive‐conserving in treating Chinese LCBM.	^492^
RT	Bodensohn, Raphael et al.	DRKS00014694	2023	Prospective, nonrandomized controlled trial	BM (NSCLC and melanoma)	SRS vs. WBRT	110	SRS cohort: mOS: 10.4m; miPFS: 7.1m; survival rates at 12 and 24 months: 48.2 and 33.4%. WBRT cohort: mOS: 6.5m; miPFS: 5.9m; survival rates at 12 and 24 months: 35.9 and 19.6%	Most notable grade 2 AEs: seizure and neurocognitive decline. No grade 3 toxicities were observed in the SRS‐cohort.	This trial did not meet its primary endpoint as the OS‐improvement of SRS compared with WBRT was nonsignificant and thus superiority could not be proven.	^129^
RT	Yang, Jonathan T et al.	NCT04343573	2022	II	NSCLC and BC with LM/other solid tumors	IFRT vs. pCSI	98	pCSI group: iPFS: median 7.5m; OS: 9.9m. IFRT group: iPFS: 2.3m; OS: 6.0m	pCSI group, grade 3 nonhematologic toxicities: fatigue, pain, and vomiting. IFRT group, grade 3 nonhematologic toxicities: fatigue, gait disturbance, and headache. No grade 5 toxicity.	Compared with photon IFRT, we found pCSI improved iPFS and OS for patients with NSCLC and BC with LM with no increase in serious TAEs.	^493^
RT	Katsuyuki Suzuki et al.	JNETS0301 (UMIN000010934)	2021	II	T1‐2N0‐1 NSCLC patients with brain oligometastasis	Surgery/SRS	18	5‐year OS: 31.8%	NA	For patients with suspected BM associated with NSCLC, bifocal local treatment could be an acceptable therapeutic strategy, especially for solitary BM.	^494^
**RT combination**											
RT + ICI	Altan, Mehmet et al.	NCT02696993	2023	I/II	NSCLC with BM	Brain SRS and systemic therapy with nivolumab and ipilimumab	13	Median number of BMs treated with SRS: 3; intracranial disease control; estimated 4‐month intracranial PFS rate: 70.7%; estimated 12‐month intracranial PFS rate: 35.4%; intracranial event‐free survival in 4 and 12 months: same at 65.5%; intracranial ORR: 38%; mOS: not reached. Extracranial disease control: ORR for extracranial disease: 25%; median time to extracranial progression: 3.8m	Most common grade 3 AEs: elevated ALT. Most common grade 4 AEs: cerebral edema. One intracranial DLT: grade 3 seizure.	Concurrent brain SRS with nivolumab/ipilimumab was safe for patients with active NSCLC BM. Preliminary analyses of treatment efficacy were encouraging for intracranial treatment response.	^495^
RT + radiotherapy enhancer	Camille Verry et al.	NANO‐RAD (NCT02820454)	2021	I	NSCLC with measurable BM	AGuIX nanoparticles + WBRT	15	13/14 evaluable patients had a clinical benefit.	Most common nonserious AEs: nervous system disorders.	Combining AGuIX with radiotherapy for patients with BM is safe and feasible.	^496^
**Breast cancer**											
Monoclonal antibody (mAb)	Kumthekar, Priya U et al.	NCT01325207	2023	I/II	I: any HER2+ histology LM; II: HER2+ BC with LM	Intrathecal trastuzumab	34	I: maximum‐tolerated dose: 80 mg; II: total patients: mPFS: 2.2m; mOS: 8.3m; HER2+ patients: mPFS: 2.8m; mOS: 10.5m	Common AEs: headache, grade 1 of nausea, grade 1 emesis, meningismus.	This study suggests promise for potentially improved outcomes of HER+ LMD patients when treated with intrathecal trastuzumab while remaining safe and well tolerated for patients.	^497^
mAb	Oberkampf, Florence et al.	NCT01373710	2023	II	HER2+BC with LM	Intrathecal trastuzumab	19	Free from neurological progression at 8 weeks: 74%; median LM‐related‐PFS: 5.9m; mOS: 7.9m	Main QLQ C30 symptoms: fatigue, pain, and sleeping disorder. No toxicity above grade 3.	Weekly administration of 150 mg of IT trastuzumab as a valuable option for HER+ BC patients with LM.	^498^
ADC	Pérez‐García, José Manuel et al.	DEBBRAH (NCT04420598)	2023	II	HER2+ or HER2‐low BCBM	T‐DXd	21	Cohort 1 (HER2+ advanced BC patients with nonprogressing BMs after local therapy): 16‐week PFS: 87.5%; ORR: 80%; Cohort 2 (asymptomatic untreated BMs): intracranial ORR: 50.0%; ORR: 50%; Cohort 3 (progressing BMs after local therapy): intracranial ORR: 44.4%; ORR:66.7% Overall intracranial ORR: 46.2%; ORR:66.7%	Common AEs: fatigue, nausea, neutropenia, and constipation.	T‐DXd showed intracranial activity with manageable toxicity and maintained the quality of life in pretreated HER2+ advanced BC patients with stable, untreated, or progressing BMs.	^499^
ADC	Bartsch, Rupert et al	TUXEDO‐1 (NCT04752059)	2022	II	HER2+ BCBM	T‐DXd	15	Intracranial ORR: 73.3%; mPFS: 14m; intracranial CR: 13.3%; partial IRR: 60%; SD: 20%; best overall IRR: 73.3%	Main grade 1/2 hematological toxicities: anemia and neutropenia. Grade 1/2 nonhematological AEs: fatigue, nausea, alopecia, constipation, hypokalemia, diarrhea, bone pain dyspnea, fall, urinary tract infection, vomiting.	T‐DXd showed a high intracranial response rate in patients with active BM from HER2+ BC.	^500^
Chemo	Sachdev, Jasgit C et al.	NCT01770353	2021	I	Metastatic BC with or without active BM	Liposomal irinotecan	10	CNS cohort: ORR 30%, clinical benefit rate: 50%. Best OR: PR in 30.0%, SD in 30.0%, PD in 20.0%	Common grade 3 or higher TEAEs: diarrhea, nausea, fatigue, asthenia, and hypokalemia. No treatment‐related TEAEs resulted in death.	Liposomal irinotecan monotherapy demonstrated antitumor activity in heavily pretreated patients with metastatic BC, with or without BM.	^501^
Chemo	Xie, Yizhao et al.	NCT02687490	2021	II	Metastatic BC with BM visceral metastases	Nab‐paclitaxel	5	PFS: 2.8m	Most common treatment‐related, grade ≥3 toxicities: neutropenia and sensory neuropathy.	Patients with BM showed poorer outcome compared with others. For patients with BM, further alternatives might be required.	^502^
Immunotherapy	Brastianos, Priscilla K et al.	NCT02886585	2024	II	BM of solid tumor	Pembrolizumab	BC (*n* = 35)	mOS: 8.0m; miPFS: 1.6m; iPFS rate at 12 weeks and 18 weeks: 44 and 26%; overall intracranial benefit rate: 42.1%; median time to progression of CNS: 1.6m; median time to progression of CNS for patients with intracranial benefit: 4.1m	Most common AEs: fatigue, nausea, headache, vomiting, transaminitis.	Programmed cell death protein 1 blockade may benefit a select group of patients with BMs.	^503^
**Combination therapy**											
mAb + mAb	Lin, Nancy U et al.	PATRICIA (NCT02536339)	2021	II	HER2+ BCBM	Pertuzumab + high‐dose trastuzumab	39	Intracranial ORR: 11%; clinical benefit rate at 4 months and 6 months: 68 and 51%	Most common TRAEs: diarrhea and fatigue. No grade 5 AEs. No new safety signals.	High‐dose trastuzumab for HER2+ CNS metastases may warrant further study.	^504^
ADC + chemo	Jenkins, Sarah et al.	NCT03190967	2023	I	HER2+ BCBM and were within 12 weeks of WBRT, SRS, surgery	T‐DM1 + TMZ	12	Median follow‐up: 9.6m; new parenchymal BM: 2 patients; tumor mutations in CSF: 6 patients	Grade 3 or 4 AEs: thrombocytopenia, neutropenia, lymphopenia, decreased CD4. No DLT.	Metronomic TMZ in combination with standard dose T‐DM1 shows low‐grade toxicity and potential activity in secondary prevention of HER2+ BM.	^136^
TKI + ADC + chemo	Lin, Nancy U et al.	HER2CLIMB (NCT02614794)	2023	II	ERBB2 + BCBM	Tucatinib + trastuzumab and capecitabine vs. placebo + trastuzumab and capecitabine	291	For all BM patients: tucatinib‐combination group: mOS: 21.6m; estimated 1‐year OS: 70.0%; estimated 2‐year OS: 48.5%; miPFS:9.9m; estimated 1‐year iPFS: 38.4%; estimated 2‐year iPFS: 19.3%; intracranial ORR: 47.3%; median intracranial DOR: 8.6m placebo combination group: mOS: 12.5m; estimated 1‐year OS: 50.6%; estimated 2‐year OS: 25.1%; intracranial DOR: 3.0m; miPFS: 4.2m; estimated 1‐year iPFS: 7.9%; estimated 2‐year iPFS: 0%; intracranial ORR: 20.0%; median intracranial DOR: 3.0m For active BM at baseline: tucatinib‐combination group: mOS:21.4m; estimated 1‐year OS:70.7%; estimated 2‐year OS: 48.9%; miPFS: 9.6m; estimated 1‐year iPFS: 32.1%; estimated 2‐year iPFS: 12.3%; placebo combination group: mOS:11.8m; estimated 1‐year OS: 46.4%; estimated 2‐year OS: 21.4%; miPFS: 4.0m; estimated 1‐year iPFS: 0%; estimated 2‐year iPFS: 0%; For stable BM at baseline: tucatinib‐combination group: mOS:21.6m; estimated 1‐year OS: 69.1%; estimated 2‐year OS: 47.8%; miPFS: 13.9m; estimated 1‐year iPFS: 52.7%; estimated 2‐year iPFS: 34.3%; placebo combination group: mOS:16.4m; estimated 1‐year OS: 57.2%; estimated 2‐year OS: 31.0%; miPFS: 5.6m; estimated 1‐year iPFS: 30%; estimated 2‐year iPFS: 0%	NA	Tucatinib in combination with trastuzumab and capecitabine improved OS while reducing the risk of developing new brain lesions.	^505^
TKI + ADC + chemo	Xie, Xiao‐Feng et al.	/	2023	II	HER2+ metastatic BC	Pyrotinib + trastuzumab and chemotherapy	15	BM: mPFS: 9.4m	Main grade 3 or 4 TRAE: diarrhea, neutropenia, leukopenia.	Pyrotinib + trastuzumab and chemotherapy offered a promising option with manageable safety profile for heavily pretreated HER2+ metastatic BC.	^506^
TKI + chemo	Yan, Min et al.	PERMEATE (NCT03691051)	2022	II	HER2+ BCBM	Pyrotinib + capecitabine	78	Cohort A (radiotherapy‐naive HER2+ BM): intracranial ORR: 74.6%; Cohort B (progressive disease after radiotherapy): intracranial ORR: 42.1%	Most common grade 3 or worse TRAE: diarrhea. No treatment‐related deaths.	Pyrotinib + capecitabine shows the activity and safety in patients with HER2+ BCBM, especially in radiotherapy‐naive population.	^198^
CDK4/6 inhibitor + mAb	Shah, Ami N et al.	/	2023	II	HER2+ BCBM	Palbociclib with trastuzumab	12	SD: 6 patients; PD: 6 patients; mPFS: 2.2m; mOS: 13.1m	Most common related AEs of any grade: lymphopenia, neutropenia, leukopenia, anemia, thrombocytopenia. No grade 5 events.	Palbociclib did not demonstrate activity in HER2+ BCBM.	^507^
RT	Phillips, Claire et al.	TROG study 16.02 (NCT02898727)	2024	II	HER2+ metastatic BC	SRS/neurosurgery	25	At 12 months: FF‐WBRT 95%; FFLF 91%; FFDBF; FFECDF 64%; OS: 96%	Most common AEs: fatigue, headache and others.	At 12 months, SRS and/or neurosurgery provided good control with low toxicity. This small study supports the practice change from WBRT to local therapies for HER2+ metastatic BCBM.	^508^
RT	Yang, Jonathan T et al.	NCT04343573	2022	II	NSCLC and BC LM	pCSI vs. IFRT	63	pCSI group: miPFS: 7.5m; OS: 9.9m; IFRT group: miPFS: 2.3m; OS: 6.0m	pCSI main grade 3 or 4 TRAE: lymphopenia, fatigue, pain, vomiting. IFRT main grade 3 or 4 TRAE: lymphopenia, fatigue, gait disturbance, headache. No difference in the rate of high‐grade TRAE between the two cohorts.	pCSI improved CNS PFS and OS for patients with NSCLC and BC with LM with no increase in serious TAEs.	^493^
RT	Yang, T Jonathan et al.	NCT03520504	2021	I	LM from solid tumors	pCSI	24	miPFS: 7.0m, mOS: 8.0m; progression‐free in the CNS for more than 12 months: 4 patients	DLTs: grade 4 lymphopenia, grade 4 thrombocytopenia, and/or grade 3 fatigue.	Hypofractionated pCSI using proton therapy is a safe treatment for patients with LM from solid tumors.	^509^
**RT combination**											
RT + chemo	Chen, Tom Wei‐Wu et al.	A‐PLUS (NCT02185352)	2024	II	BCBM	3 cycles of BEEP followed by WBRT vs. WBRT alone	118	Experimental arm: miPFS: 8.1m; intracranial ORR at 2 months: 41.9%; 8‐month iPFS: 48.7%; Control arm: miPFS: 6.5m; intracranial ORR at 2 months: 52.6%; 8‐month iPFS: 26.3%	Most common AEs in the BEEP arm: neutropenia, nausea, anemia, and leukopenia.	Induction BEEP before WBRT may improve the control of BCBM compared with using upfront WBRT.	^510^
RT + TKI	Kim, In Ah et al.	NRG/RTOG 1119 (NCT01622868)	2024	II	HER2+ BCBM	RT + concurrent lapatinib vs. RT alone	136	RT + lapatinib group: 12‐week CR: 0%; 4‐week CR rate: 1%; ORR at 12 weeks: 47%; ORR at 4 weeks: 55%; RT alone group: 12‐week CR: 6%; 4‐week CR rate: 4%; ORR at 12 weeks: 60%; ORR at 4 weeks: 42%	Most common AEs: diarrhea. No grade 5 TRAEs.	The addition of 6 weeks of concomitant lapatinib to WBRT/SRS did not improve the primary endpoint of 12‐week CR rate or 12‐week ORR. Adding lapatinib to WBRT/SRS showed improvement of 4‐week ORR, suggesting a short‐term benefit from concomitant therapy.	^511^
RT + TKI + chemo	Yang, Zhaozhi et al.	NCT04582968	2024	II	ERBB2 + BCBM	SRS + pyrotinib and capecitabine vs. WBRT + pyrotinib and capecitabine	40	1‐year iPFS rate: 74.9%; miPFS: 18.0m; 1‐year PFS rate: 66.9%; mPFS: 17.6m; Intracranial ORR: 85%; mOS: not reached	Most common grade 3 or 4 TRAEs: diarrhea.	Radiotherapy combined with pyrotinib and capecitabine is associated with long intracranial survival benefit in patients with ERBB2 + advanced BCBM with an acceptable safety profile.	^512^
RT + TKI	Morikawa, Aki et al.	NCT01724606	2021	I	BCBM	Sorafenib with WBRT	19	Maximum tolerated dose: 200mg; miPFS: 12.8m; ORR:71%	Most common TRAE: fatigue and hyperglycemia; DLT at 200 mg: grade 4 increased lipase; DLT at 400 mg: grade 3 rash.	Concurrent WBRT and sorafenib appear safe at 200 mg daily dose with clinical activity. CNS response was favorable compared with historical controls.	^513^
RT + radiosensitivity enhancer	Verry, Camille et al.	NANO‐RAD (NCT02820454)	2021	I	Patients with multiple BM (including lung cancer, melanoma, BC, colon cancer)	WBRT + AGuIX nanoparticles	15; BC (*n* = 2)	Overall miPFS: 5.5m; mOS: 5.5m	No DLTs were observed up to AGuIX 100 mg/kg.	Combining AGuIX with radiotherapy for patients with BM is safe and feasible, AGuIX specifically targets BM and is retained within tumors for up to 1 week.	^496^

Abbreviations: AE, adverse events; ALK, anaplastic lymphoma kinase; BEEP, bevacizumab, etoposide, and cisplatin; CNS, central nervous system; CR, complete response; DCR, disease control rate; DLT, dose‐limiting toxicity; DOR, duration of response; DBF, distant brain failure; ECDF, extracranial disease failure; EGFRm, epidermal growth factor receptor mutated; FF, freedom from; HER2, human epidermal growth factor receptor 2; iPFS, intracranial progression free survival; IFRT, photon involved‐field radiotherapy; IRR, intracranial response rate; LM, leptomeningeal metastasis; LF, local failure; OS, overall survival; ORR, objective response rate; OR, overall response; pCSI, proton craniospinal irradiation; PFS, progression free survival; PD, progressive disease; PR, partial response; RT, radiotherapy; SRS, stereotactic radiosurgery; SD, stable disease; WBRT, whole brain radiotherapy; T‐DXd, trastuzumab deruxtecan; T‐DM1, trastuzumab emtansine; TEAE, treatment emergent adverse events; TMZ, temozolomide; TRAE, treatment‐related adverse event; TKI, tyrosine kinase; TTP, time to progression.

STAT3 plays a critical role in promoting the progression of BM, making it a promising therapeutic target. The IL6–JAK–STAT3 axis and the HLA‐G–SPAG9–STAT3 axis have been identified as key drivers of BM progression.^376^ Melanoma and BM cells secrete IL6, leading to increased STAT3 in astrocytes which stimulates tumor growth. Inhibiting STAT3 has shown significant promise in reducing tumor proliferation.^405^, ^514^ The 2023 WCLC Congress highlighted the therapeutic potential of STAT3 inhibitors specifically for LCBM. Silibinin, a natural polyphenolic flavonoid compound, has demonstrated STAT3‐inhibiting properties and efficacy in treating LCBM.^515^ Clinical trials investigating silibinin's effectiveness in LCBM, are currently underway (NCT05689619).^516^

#### 8.1.2 TTFields

In vitro and animal experiments have demonstrated that TTFields significantly inhibit the growth of various tumors, induce cell death, and regulate immune function.^517^ In particular, for brain tumors, current animal studies have shown that TTFields can transiently open the BBB.^518^, ^519^, ^520^, ^521^, ^522^, ^523^ This temporary opening enhances drug permeability, thereby improving the efficacy of treatments against intracranial tumors. Although high‐level evidence for TTFields in treating LCBM is currently lacking, this treatment has been shown to extend the survival of GBM.^524^, ^525^, ^526^ This suggests a potential role for TTFields in treating LCBM (Table 2).

Similar to other tumors, TTFields have mild adverse reactions in LCBM, predominantly grade 1–2 skin reactions. Phase II clinical trial COMET (NCT01755624) demonstrated that TTFields had no serious adverse reactions in treating NSCLC BM, with the only reported adverse effect being mild dermatitis.^527^ Additionally, a study (NCT03903640) explored TTFields combined with ICIs (nivolumab and ipilimumab), achieving a 100% response rate for both intracranial and extracranial tumor. The median mOS, intracranial, and extracranial PFS were reported as 104.5, 99.5, and 58 days, respectively. However, this study was limited by its small sample size (only two patients), which necessitates further validation, particularly concerning the high incidence of serious adverse reactions such as death and deep vein thrombosis. The 2024 ASCO conference highlighted that TTFields combined with SRS significantly extended median intracranial progression time (TTFields + SRS vs. TTFields + best supportive care: 21.9 vs. 11.3 months).^528^, ^529^, ^530^ Nevertheless, factors such as the high cost may constrain the utilization and adoption of TTFields in clinical practice.^531^

#### 8.1.3 Antibody–drug conjugates

ADCs are a class of anticancer therapies designed to deliver cytotoxic drugs directly to tumor cells, combining the benefits of both cytotoxic chemotherapy and targeted therapy (Table 2).^532^, ^533^ The DEBBRAH trial demonstrated that the ADC trastuzumab deruxtecan, targeting HER2, effectively delays intracranial progression in BCBM.^181^, ^499^ HER3 is notably overexpressed in both LCBM and BCBM, suggesting that targeting HER3 could be a critical strategy for treatment.^534^, ^535^ Phase II clinical trial HERTHENA‐Lung01 highlighted the therapeutic efficacy of HER3‐DXd in patients who progressed after EGFR‐targeted therapy or chemotherapy.^165^ Ongoing investigations are also evaluating HER3‐DXd's role in BM (NCT05865990). Furthermore, ANG1005, a brain‐penetrating peptide–drug conjugate, has demonstrated activity in BC patients suffering from leptomeningeal carcinomatosis and recurrent BM.^536^ Sacituzumab, an ADC targeting Trop‐2, is also being investigated for its therapeutic potential (NCT06401824).

### 8.2 BCBM

#### 8.2.1 Immunotherapy

Immunotherapy is a prominent approach in treating BMs and has extended survival in various tumors.^537^, ^538^ However, BCBM, particularly “cold” tumors, shows low PD‐L1 expression (positive rate of only 25% in TNBC), limiting the success of current immunotherapies.^304^, ^539^ Research is now focusing on enhancing immunotherapy for TNBC and improving the BM microenvironment.^540^ For instance, doxorubicin induces senescence in intracranial BC cells and stimulates CD8+ T cell antitumor responses, thereby boosting the effectiveness of anti‐PD‐1 therapies in BCBM.^541^ Nanoparticles and nanomaterials, such as SIL@T, can induce immunogenic cell death and reverse the immunosuppressive microenvironment, offering a promising strategy.^542^, ^543^ CAR‐T therapy, which combines T cell cytolytic activity with antibody specificity, shows potential treatment strategies. Particularly, EGFR806 CAR‐T targeting the highly expressed EGFR in BCBM has demonstrated strong antitumor effects in mouse models.^544^ CAR‐NK cell‐derived exosomes also show therapeutic promise.^545^ Additionally, bioinformatics analyses by Najjary et al.^332^ suggest that LAG3–LGALS3, TIGIT–NECTIN2, and VTCN1 could be promising targets for immunotherapy.^304^

#### 8.2.2 Targeting the BBB

Modifying the structure of existing drugs and the BBB, or using drug carriers can significantly enhance drug concentrations within the brain.^546^ Zuclopenthixol, which binds to the juxtamembrane region of HER2, effectively inhibits the growth of BC cells.^547^ NEO100, a high‐purity form of the natural monoterpene perillyl alcohol, not only enhances BBB permeability but also increases the delivery of trastuzumab to the brain.^548^ Angeli et al.^549^ designed a trastuzumab Fab fragment to reduce its extravasation through cerebral blood flow and improve brain permeability. Additionally, employing an adenovirus‐associated virus vector for intrathecal administration of trastuzumab has shown significant potential in inhibiting tumor growth.^550^ Combining trastuzumab with biocytin‐TMR can markedly increase intracranial drug concentration and reduce drug elimination rates.^551^ Physical techniques also offer promising approaches to enhance BBB permeability and treat intracranial tumors. For instance, low‐intensity “whole‐brain” ultrasound combined with VCAM‐1 functionalized microbubbles can open the BBB and improve drug delivery.^552^, ^553^ TTFields therapy, utilizing moderate frequency (100–300 kHz) and low‐intensity (1–3 V/cm) alternating electric fields, has recently been shown to temporarily open the BBB and inhibit tumors.^248^, ^517^, ^531^

New applications of existing drugs and the development of novel therapeutic agents, including nanoparticles, peptides, proteases, and new inhibitors, offer promising advancements.^122^, ^554^ Atovaquone, an antiprotozoal drug used for pneumocystis pneumonia, has been shown to inhibit BM by decreasing integrin α6, integrin β4, FAK, Src, and Vimentin expression.^555^ Preclinical studies suggest that medicinal mushroom blends can effectively suppress cerebellar metastasis in BC.^556^ Dual‐action or dual‐target nanotechnology not only enhances BBB permeability but also modulates tumor cells and the microenvironment to improve tumor eradication.^557^, ^558^ The endothelial WNT signaling pathway and pericytes are crucial for maintaining BBB integrity. For example, Mu et al.^559^ utilized ICAM‐1‐targeted nanoparticles (NI@I‐NPs) coloaded with nitazoxanamide and ibrutinib to disrupt the BBB and enhance chemotherapy efficacy. Strategies targeting the mitochondrial genome can increase BBB penetration of nanoparticles, allowing for effective treatment of BM.^560^ Similarly, VCAM‐1‐targeting nano‐wogonin (W@V‐NPs) inhibits WNT signaling at the BTB, disrupts lipogenesis, and impairs the BC cells' ability to adapt to the intracranial low‐lipid microenvironment, thereby restricting tumor growth.^561^

The dual‐acting peptide PepH3–vCPP2319, which combines the specific anti‐BC peptide (vCPP2319) with a BBB shuttling peptide (PepH3), has demonstrated effective BBB penetration in both in vitro/vivo models.^562^ Additionally, the interaction between stromal cells and tumor cells promotes the formation of glycocalyx, potentially leading to resistance to HER2‐targeted therapies such as neratinib. Mucinase that inhibits the EGFR/HER2 signaling pathway has been shown to reverse treatment resistance.^563^ BRBP1‐modified multifunctional liposomes are designed to actively target Twinfilin 1 and overcome paclitaxel resistance.^564^ Customized micelles, such as those based on the in situ microenvironment (T‐M/siRNA), can simultaneously deliver siRNA and chemotherapeutic agents to tumor cells. Meanwhile, it inhibits astrocyte activation and the immunosuppressive activation of microglia, thereby enhancing antitumor effects.^565^ Micelles modified with Angiopep‐2, known as Ang‐MIC‐PTX/LP, offer high drug loading efficiency for paclitaxel and lapatinib, and their combination has proven effective in killing intracranial tumor cells.^566^

#### 8.2.3 Targeting tumor metabolism

Lipogenesis enables tumor cells to adapt to the low‐lipid brain microenvironment, facilitating colonization and growth.^236^ Brain‐tropic tumor cells can autonomously produce fatty acids by upregulating lipogenic fatty acid synthases, which is not observed in extracranial metastases. This differential metabolic adaptation may explain organ‐specific metastatic patterns.^567^ Targeting lipid metabolism in BC presents a promising therapeutic approach. For instance, the fatty acid synthase inhibitor TVB‐2640, in combination with the topoisomerase inhibitor SN‐38, has shown synergy in treating BM of TNBC.^568^ ACSS2, a crucial regulator of fatty acid synthesis and protein acetylation, can be inhibited by brain‐permeable ACSS2 inhibitors. Furthermore, ACSS2 inhibitors work synergistically with radiation to block BCBM.^569^ Monoacylglycerol lipase (MAGL) regulates the degradation of 2‐arachidonoylglycerol and promotes inflammatory factor production. Consequently, the MAGL inhibitor AM9928 has been shown to inhibit the adhesion and transendothelial migration of BC cells.^570^ Despite the effectiveness of some lipid metabolism regulators in preclinical models, transitioning these therapies from the laboratory to clinical practice remains a significant challenge.^571^, ^572^

HER3, a receptor tyrosine kinase with oncogenic properties, is highly expressed in a significant proportion of patients with BMs (72.9% in LC and 75.0% in BC). Targeting HER3 with hyperbranched polymers carrying doxorubicin has proven effective in increasing drug concentration at the metastatic site.^573^ Similar to HER2, HER3 could be a viable target for ADC.^534^ Additionally, stem cell‐mediated bifunctional therapies targeting EGFR and DR4/5 have shown effectiveness in inhibiting perivascular niche micrometastasis and leptomeningeal metastasis.^574^ The AR, expressed in most BCBM, also represents a potential therapeutic target.^575^

## 9 CONCLUSION

LC and BC are prominent causes of BM.^576^, ^577^ While the diagnosis of BM is now routine, challenges remain in treatment and maintaining quality of life.^3^ Advances in diagnostic and therapeutic technologies have significantly improved the prognosis for LCBM patients, and long‐term survival is now increasingly feasible for BCBM patients.^578^ Understanding the influence of molecular subtypes and other factors on prognosis is crucial for achieving precision treatment and identifying the most responsive patient populations.^189^, ^232^, ^579^, ^580^, ^581^ Current treatment strategies for BM typically involve a combination of local and systemic therapies. For LC, targeted therapies, immunotherapies, and RT have transformed the management of NSCLC patients with BM, while SCLC is primarily treated with chemotherapy and RT. In contrast, treatment for BCBM involves ADC, monoclonal antibodies, TKIs, chemotherapy, and RT. Recent advancements have reversed the previously poor prognosis for BM patients.^582^ A decade ago, these patients were often excluded from clinical trials due to poor prognosis, but recent recommendations advocate for their inclusion under nonexclusion criteria (ASCO/FoCR/FDA task force recommendation).^237^ Prospective clinical trials are primarily focused on LC, BC, and melanoma.^583^ The development of TKIs such as osimertinib, icotinib, and pyrotinib, as well as monoclonal antibodies like trastuzumab, pembrolizumab, and nivolumab, represents a significant area of interest for systemic therapy.^583^ Although leptomeningeal metastasis historically has a poor prognosis, recent improvements in diagnosis and treatment have led to better outcomes.^584^ Furthermore, recognizing the brain's role in behavior and emotions underscores the importance of preserving neurocognitive function and quality of life alongside survival (NCT04890028).^585^ Efforts by oncologists highlight the need for continued research into optimizing current treatment and developing new diagnostic and therapeutic approaches for BM. Emerging diagnostic methods, including less invasive circulating biomarkers, offer promise for the management of BMs.^576^ CSF testing is increasingly used for diagnosing and predicting treatment outcomes.^586^ While ctDNA has proven useful in various tumors, its detection rate in leptomeningeal metastases requires improvement.^261^ Despite these challenges, liquid biopsy remains a promising tool for tumors, facilitating diagnosis, prognosis, and monitoring of minimal residual disease and recurrence.^385^

In BM, AI is increasingly integrated with multimodal data, including imaging omics and pathology omics, to enhance predictions of metastasis occurrence and prognosis, identify primary tumors and their molecular subtypes, and assist in RT planning.^587^, ^588^ AI applications in tumor models are advancing high‐throughput systems capable of real‐time modeling and monitoring of tumor development and biophysical characteristics.^589^ Challenges such as the difficulty in obtaining samples for BM research can limit the robustness of evidence.^590^ However, AI‐driven big data platforms, which combine imaging omics, pathology omics, and transcriptomics, are emerging to facilitate the exploration of clinical diagnosis, treatment, and the mechanisms underlying BM.^591^, ^592^ Integrating bulk and single‐cell transcriptome helps uncover the molecular mechanisms associated with tumor development and provides a comprehensive understanding of the TME.^304^ Recent advancements in transcriptomics, proteomics, genomics, metabolomics, and methylation sequencing have provided multidimensional data for understanding BM.^323^, ^341^, ^346^ These developments significantly contribute to further elucidation of the BM complexities.^261^

Preclinical research in BM has made significant strides, particularly with the use of brain‐tropic cell lines and animal models.^242^ Recent innovations, such as organoid models, have introduced breakthroughs in basic research and personalized treatment strategies. Additionally, hydrogel models that simulate tumor dormancy and interactions with the microenvironment, as well as microfluidic chips that mimic the metastasis process, represent cutting‐edge tools that warrant further investigation.^278^, ^279^, ^280^, ^281^ However, each model comes with its own set of advantages and limitations in BM research.^261^ Understanding the key carcinogenic steps in the tumor‐soil theory is crucial for developing effective therapeutic targets. This includes elucidating how tumors escape the primary site, protect themselves during dissemination, interact with brain microenvironment components, and the nature of the brain microenvironment once metastasis occurs.^593^, ^594^ Currently, treatment strategies predominantly focus on the primary tumor, with relatively few therapies developed specifically for BMs.^277^, ^372^, ^373^, ^418^, ^424^, ^427^ Specifically targeted treatments based on the mechanisms of BM are still limited. Effective treatment for BM often requires overcoming the BBB to increase intracranial drug concentrations.^546^ Therefore, the development of TKIs and nanotherapy that can penetrate the BBB are promising directions for future research.^197^, ^595^, ^596^, ^597^ While addressing BMs is essential, it is equally important to consider the primary tumor. Comparing the similarities and differences in metastases from various primary tumors can provide valuable insights for precise therapy.^598^, ^599^

The National Cancer Institute Collaborative Workshop has emphasized the need to establish research priorities that address critical areas of unmet need for BM patients.^600^ Key areas requiring attention include identifying and screening high‐risk patients, evaluating physical and cognitive impairments, addressing discrepancies between preclinical models and clinical realities, and developing BM‐specific diagnostic tests and treatment strategies.^276^, ^600^ Over the past decade, multidisciplinary team collaborations have significantly improved the prognosis for BM patients.^601^, ^602^ Continued focus on these research priorities will advance our understanding and management of BMs, highlighting the potential for achieving substantial progress and improving outcomes in the future.

## AUTHOR CONTRIBUTIONS

G. T. and L. C. reviewed the literatures and wrote the manuscript. J. N., W. X., C. W., and G. X. were responsible for discussing the manuscript and making critical revisions to the logic and grammar. J. N., C. W., G. T., and L. C. drafted and polished the figures and tables. J. Y. and R. Z. provided financial support. G. T. and L. C. contributed equally to the first author while J. Y. and R. Z. were equated to the corresponding author. All authors have reviewed and approved the publication of the manuscript.

## CONFLICT OF INTEREST STATEMENT

The authors declare that there is no conflict of interest in any aspect.

## ETHICS STATEMENT

Not applicable.

## Data Availability

Not applicable.
